# NMR Unveils Activity Mechanism of Linear Spider Venom Peptide Fragments Selected by Neural Networks Against Staphylococci Including MRSA

**DOI:** 10.3390/pharmaceutics17121526

**Published:** 2025-11-27

**Authors:** Pavel A. Mironov, Anna A. Baranova, Vera A. Alferova, Natalya S. Egorova, Anastasia A. Ignatova, Alexey V. Feofanov, Zakhar O. Shenkarev, Peter V. Dubovskii

**Affiliations:** 1 Shemyakin-Ovchinnikov Institute of Bioorganic Chemistry, Russian Academy of Sciences, 16/10 Miklukho-Maklaya str., Moscow 117997, Russia; pavel.mironov@student.msu.ru (P.A.M.); anjabaranowa@list.ru (A.A.B.); alferovava@gmail.com (V.A.A.); natalyegorov@yandex.ru (N.S.E.); avfeofanov@yandex.ru (A.V.F.); zakhar.shenkarev@nmr.ru (Z.O.S.); 2 Bioengineering Department, Faculty of Biology, Lomonosov Moscow State University, 1 Leninsky Gory str., Moscow 119234, Russia; 3 Moscow Center for Advanced Studies, 20 Kulakova str., Moscow 123592, Russia

**Keywords:** spider venom, linear membrane-active peptide, methicillin resistant *S. aureus* (MRSA), neural network, deep-learning, hemolytic activity, chemical synthesis, high resolution NMR spectroscopy, wide-line NMR spectroscopy, selectivity mechanism

## Abstract

**Background/Objectives:** Methicillin-resistant *Staphylococcus aureus* (MRSA) poses a significant global health threat due to its increasing resistance to conventional antibiotics. Antimicrobial peptides (AMPs) derived from natural sources represent a promising alternative. Fragments of spider membrane-active toxins can serve as AMPs with anti-MRSA activity. **Methods:** To demonstrate this, amino acid sequences of approximately 2000 linear spider venom peptides were fragmented into 9–22-residue-long moieties (75,235 in total) and pre-trained neural networks were used to predict their anti-MRSA activity. As many as 15 peptides with high predicted activity were synthesized, and three AMPs with high anti-MRSA and low hemolytic activities were selected. One of these peptides was studied using high-resolution ^1^H-, ^13^C-, and ^15^N-NMR spectroscopy in an aqueous solution and lyso-palmitoylphosphatidylglycerol (LPPG) micelles. Wide-line ^31^P-NMR was applied to multilamellar phospholipid liposomes composed of phosphatidylcholine (PC) or phosphatidylglycerol (PG). **Results**: Low hemolytic activity is explained by non-specific interaction with PC whereas high antibacterial activity arises from specific interaction with PG accompanied with the formation of a tight complex between the *N*-terminal tripeptide fragment and PG headgroup. The structure of a such complex, stabilized by an ionic interaction between the *N*-terminal NH_3_^+^ group and the lipid phosphate, was determined based on peptide–LPPG NOEs. The most favorable ratio between anti-MRSA and hemolytic activities, i.e., selectivity of the peptides, is attained when the tripeptide consists exclusively of phenylalanine and tryptophan residues. Confocal microscopy confirmed that the most selective peptide deteriorates the plasma membrane of *S. aureus*. **Conclusions**: This approach may enable the production of highly selective AMPs against *Stapylococci*, including MRSA.

## 1. Introduction

The discovery and use of antibiotics in the 20th century were a lifesaver for humanity. However, due to their widespread use, resistant microorganisms have emerged. A group of bacteria called ESKAPE (*Enterococcus faecium*, *Staphylococcus aureus*, *Klebsiella pneumoniae*, *Acinetobacter baumannii*, *Pseudomonas aeruginosa*, and *Enterobacter* spp.) has emerged, requiring the development of new antibacterial drugs against them, because the traditional antibiotics became ineffective [1].

Intensive efforts are focused on developing antimicrobial peptides (AMP) against methicillin-resistant *Staphylococcus aureus* (MRSA), a major global health threat responsible for a range of infections from superficial skin conditions to invasive sepsis. Since bacterial resistance to AMP based on membrane-active toxins develops slowly, these peptides could play a decisive role here [2]. The development of such AMP is progressing rapidly. For example, Wang G. and co-workers are developing anti-MRSA AMP based on the human cathelicidin LL-37 [3,4,5]. Melittin from bee venom has demonstrated efficacy against MRSA biofilms [6]. Peptides derived from the skin secretions of the Australian frog have been shown to suppress the growth of MRSA skin infections in mice and do not induce bacterial resistance to them [7]. Toxins, such as a tetradecapeptide from wasp venom, exhibit strong anti-MRSA activity and low cytotoxicity [8]. Toxins from spider venom can also be utilized as a source for such AMP. Among them, linear membrane-active peptides are of particular interest. It is believed that long evolution of the spiders in harsh environments provided their venoms with especially potent toxins possessing antimicrobial and cytolytic properties [9]. Examples such as LyeTx-I, a peptide from *Lycosa erythrognatha* venom, exhibit good anti-MRSA activity [10]. Latarcins from *Lachesana tarabaevi* spider venom exhibit broad-spectrum antimicrobial potency [11]. They were studied by a number of biophysical techniques to establish structure-activity relationships [12,13]. One of the peptides, latarcin 1 exhibited interesting membrane-active properties [14]. It caused dye leakage from liposomes composed of DOPG. In case of the liposomes composed of DOPG/DOPE (1:1) mixture, no dye leakage was observed. Probably, this is due to specific interaction of the peptide with the head group of DOPE molecule preventing the peptide molecule from embedding in the membrane. However, this hypothesis has not been proven experimentally. Instead, spatial structure of latarcin 1 was studied in perdeuterated SDS micelles only [15].

In this study, we present an approach that integrates a library of linear peptide (LP) fragments derived from spider venom with advanced neural network prediction models to discover novel AMP effective against MRSA. By synthesizing and conducting detailed structural and functional characterization of the resulting AMP, we elucidated the key determinants of their antibacterial potency and selectivity. Structural insights based on NMR spectroscopy and confocal microscopy reveal the mechanisms of AMP’s membrane perturbation and structural features governing the membrane selectivity, thereby advancing the development of spider venom-derived AMP as next-generation antibiotics against MRSA. Importantly, numerous previous studies were based on NMR of AMP in perdeuterated commercial detergents, either SDS, or DPC. However, the structure of the peptides depends on the environment. Detergents mimicking the composition of the bacterial plasma membrane should be used. In the current work we determined the structure of one of the active peptides (designated IX) in LPPG micelles, which mimic the environment of cytoplasmic membrane of *S. aureus*, including MRSA. The structure prediction methods such as AlphaFold [16] are also of limited value unless they predict structure in the appropriate environment.

## 2. Materials and Methods

### 2.1. Peptide Synthesis

Peptides were synthesized by standard Fmoc solid-phase peptide synthesis on TCP-Resin (Tritylchloride-Polystyrene-Resin, Intavis, Germany) using HATU/DIPEA coupling chemistry and subsequently cleaved with TFA/DTT/H_2_O/TIS. Purification was performed by preparative reverse-phase HPLC on a YMC Actus Triart C18 (10 µm, 30 × 150 mm) and verified by UPLC–MS (Thermo Finnigan LCQ Deca XP Plus, Waltham, MA, USA), yielding products with over 95% purity, (Table S2, see Supplemental Methods). All solvents and reagents were used without additional purification.

### 2.2. Database Assembly

**Figure 1:**
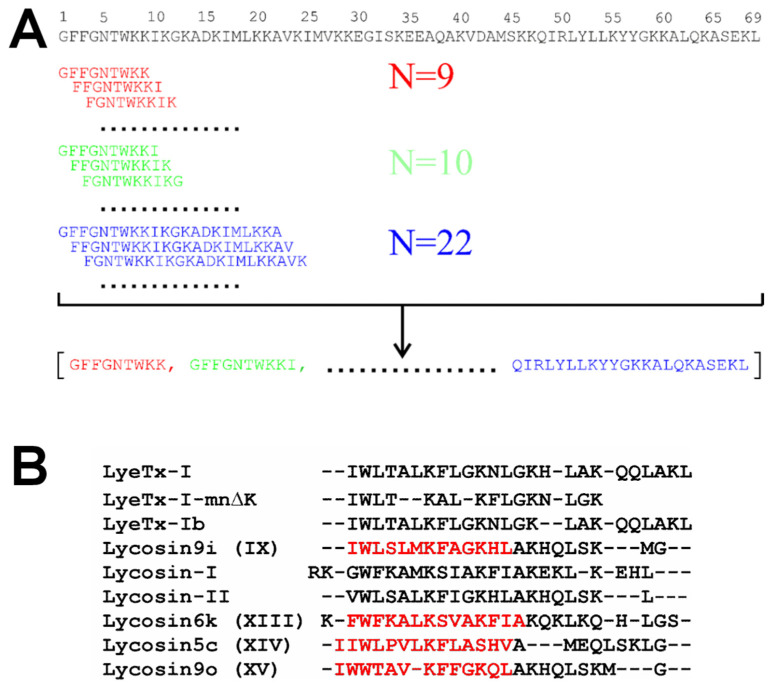
Fragmentation and alignment of amino acid sequences of linear peptides (LP) from spider venom. (**A**) Fragmentation of 69-residue cytinsectotoxin-1a (sequence shown above) into fragments ranging from 9 to 22 residues in length (shown in different colors). (**B**) Alignment of LPs from spider venoms that are parental to peptides IX, XIII, XIV, and XV (in red) with homologous spider LPs and their analogues.

All LP from spider venoms were gathered from literature sources [9,12]. The peptide fragmentation procedure was implemented as a Python (version 3.10.16) program. Its algorithm is illustrated in Figure 1A. Database of LP ‘active’ and ‘inactive’ against MRSA used to train neural networks were prepared from AMP databases such as APD3 [17], DBAASP [18], DRAMP [19], and from recent literature, as not all latest findings are promptly integrated into the databases. Additional details can be found in the Section 3.1 and Supplemental Data (Table S1).

### 2.3. High Resolution NMR Spectroscopy

NMR experiments in aqueous and micellar solutions were performed using samples containing 0.2–1.0 mM of peptide IX in 5% D_2_O at pH of 4.5. To model a membrane environment, 1-palmitoyl-2-hydroxy-sn-glycero-3-phospho-(1′-rac-glycerol) (LPPG, 16:0 Lyso PG, Avanti, Alabaster, AL, USA) was added stepwise to the aqueous peptide IX sample using a concentrated stock solution until a detergent-to-peptide (D:P) molar ratio of 140:1 was achieved. The NMR spectra were acquired on a Bruker Avance III 800 MHz spectrometer (Karlsruhe, Germany) equipped with a room-temperature triple-resonance (^1^H, ^13^C, ^15^N) probe. Almost complete ^1^H, ^13^C, and ^15^N resonance assignments for peptide IX in aqueous solution and LPPG micelles were obtained via a combination of 2D ^1^H-TOCSY (τ_m_ = 60 ms), ^1^H-NOESY (τ_m_ = 150 ms) and natural abundance ^13^C-HSQC and ^15^N-HSQC spectra measured at 5 °C and 45 °C, respectively, using the CARA 1.9.1.7 software (http://cara.nmr.ch/doku.php/Home (accessed on 12 December 2024)). The ^3^J_H_^N^_H_^α^ scalar couplings were estimated via line-shape analysis in 2D TOCSY and NOESY spectra. The temperature gradients of amide protons (∆δ^1^H^N^/∆T) were measured from a series of 2D TOCSY spectra in water and 2D NOESY spectra in LPPG acquired in the temperature range of 15–45 °C. The pH-dependences of the ^1^H^N^ and ^1^H^δ2^ His12 chemical shifts were measured in the pH range 2–9 from a series of 2D TOCSY spectra in water at 30 °C and 2D NOESY spectra in LPPG micelles at 45 °C. The pKa values were calculated as previously [20].

Secondary structure and random-coil indices (RCIs) predictions for peptide IX were derived from ^1^H, ^13^C, and ^15^N chemical shifts using TALOS-N [21]. Distance constraints were obtained from cross-peak intensities in 2D NOESY spectra (τ_m_ = 150 ms) and φ angle restraints were obtained from ^3^J_H_^N^_H_^α^ couplings. Assuming that HN groups with ∆δ^1^H^N^/∆T > −4.5 ppb/K participate in the hydrogen bonds formation, hydrogen bond restraints were applied. The 3D structures were calculated using CYANA ver. 3.98 [22].

To investigate the topology of the peptide in the micelle, the paramagnetic probe of 16-doxylstearic acid (16-DSA) was added to the peptide IX sample in LPPG micelles (D:P of 140:1) at a 16-DSA:peptide ratio of ~1:1 (approximately one probe per micelle). The intensities of intraresidual H^α^–H^N^ or H^β^–H^N^ NOESY cross-peaks were measured to calculate relative attenuation induces by the spin label. Residues with attenuation values below the threshold of 0.5 were considered to be immersed in the lipid phase, thereby classified as being ‘inside’ the micelle, while those above were classified as being ‘outside’.

To interpret residue-level attenuation, an inclusion function *μ*(*p*_0_, *R*) was defined to quantify the position of a rigid peptide molecule relative to a micelle of radius *R* centered at point *p*_0_ *=* (*x*_0_, *y*_0_, *z*_0_):

(1) $$\mu \left(p_{0},R\right)=\sum_{i}\max\left(0,A_{i}\cdot \left(R-d_{i}\right)\right)$$

where summation is over all residues of the peptide, *i* is the number of the current residue, $d_{i}=\left\|p_{i}-p_{0}\right\|$ is the Euclidean distance between the micelle center and the H^α^ atom of the *i*-th residue having coordinates *p_i_* = (*x_i_*, *y_i_*, *z_i_*), and *A_i_* is the weighting factor for the *i*-th residue defined by attenuation-based classification, with *A_i_* = −1 for residues ‘inside’ the micelle (attenuation below threshold), and *A_i_* = +1 for residues ‘outside’ (attenuation above threshold).

This inclusion function has a zero minimum value only when all H^α^ atom assigned to being ‘inside’ or ‘outside’ the micelle are indeed located ‘inside’ or ‘outside’ a sphere of radius *R* with center *p*_0_. The inclusion function was minimized using optimize module from SciPy python library, which yielded ‘optimal’ values of *R* and *p*_0_.

Distance constraints between the peptide and detergent protons derived from the cross-peak intensities in the 2D NOESY spectrum (τ_m_ = 150 ms) were used to calculate the structure of the peptide/LPPG complex.

### 2.4. Wide-Line ^31^P-NMR Spectroscopy

^31^P NMR spectra were obtained on Bruker Avance-III 600 MHz spectrometer equipped with a broadband probehead. ^31^P-NMR spectra were recorded with parameters, described in [23]. Theoretical ^31^P-NMR spectra were calculated and fitted to the experimental ones using program P-Fit [24].

The multilamellar vesicles (MLVs) composed from either 1,2-dioleoyl-sn-glycero-3-phosphocholine (DOPC), or 1,2-dioleoyl-sn-glycero-3-phosphoglycerol (DOPG) were used (Avanti Polar Lipids, Alabaster, AL, USA). For sample preparation, 7 mg of phospholipid powder was dispersed in 200 µL of D_2_O (99.9%), followed by mechanical agitation to ensure homogeneity. Peptide solutions in Tris-HCl buffer (pH 7.0) were added to achieve the desired lipid-to-peptide (L:P) molar ratios.

### 2.5. NMR Diffusion Measurements

The translational diffusion coefficients for peptide IX and LPPG micelles were measured using PGSTE-WATERGATE pulse sequence [25] with diffusion delay of 200, 300 and 400 ms. The measurements were conducted on Bruker Avance III 800 MHz spectrometers equipped with a room-temperature triple-resonance probe at 30 °C. The diffusion coefficients measured in the micellar solutions were corrected for excluded volume effects arising from the presence of micelles, which occupy a non-negligible fraction of the sample (see Supplemental Methods):

(2) $$D_{f}=D_{f}^{0}(1-3.6\cdot \omega )$$

where $D_{f}$ is the experimentally determined diffusion coefficient, and $D_{f}^{0}$ represents the true diffusion coefficient, in the conditions where interparticle interactions are minimized. ω denotes the volume fraction occupied by the diffusing species [26,27]. The volume of detergent micelles was calculated assuming the density of approximately 1 g/cm^3^.

From the corrected translational diffusion coefficient, the hydrodynamic radius of the diffusing entities was estimated using the Stokes-Einstein relationship:

(3) $$D_{f}^{0}=\frac{kT}{6\pi \eta R_{H}}$$

where η is the dynamic viscosity of the solvent (water), $R_{H}$ is the hydrodynamic (Stokes) radius, *T* is the absolute temperature, and *k* is Boltzmann’s constant.

### 2.6. Testing Biological Activity of Peptides and Confocal Microscopy Measurements

Hemolytic activity was assessed using fresh capillary blood from a human volunteer, as described in [28]. Each peptide’s hemolytic activity was tested in two independent experiments.

For antimicrobial activity determination, Gram-positive strains (*Staphylococcus aureus* 209P, ATCC 29213, ATCC 25923, ATCC 43300 [MRSA], *S. epidermidis* ATCC 14990) were used. Experiments were conducted at bacterial concentrations of (0.5–1) × 10^5^ cells/mL. The minimum inhibitory concentration (MIC), defined as the lowest concentration that fully inhibits bacterial growth, was determined by two-fold serial dilutions. Briefly, overnight cultures in Mueller-Hinton broth (MHB, HiMedia, Mumbai, Maharashtra, India) were diluted 1:100 into fresh MHB and incubated at 37 °C for 3 h. Cultures were then adjusted to ~1 × 10^5^ cells/mL (by OD600) and used as an inoculate. Peptide stocks (10 mg/mL in water) were prepared in MHB to achieve a starting concentration of 200 μg/mL and subsequently diluted twofold.

Confocal laser scanning microscopy (CLSM) measurements were performed with an SP2 confocal inverted microscope (Leica Microsystems GmbH, Wetzlar, Germany) using the water-immersion 63×, 1.2 NA HCX PL APO objective. A typical voxel size was 0.15 × 0.15 × 0.9 μm. Excitation wavelengths were 561 nm for propidium iodide (PI) and 488 nm for 2′,7′-Bis-(2-carboxyethyl)-5-(and-6)-carboxyfluorescein (BCECF) (Sigma-Aldrich, MO, USA). Laser power was 3–10 μW the sample. Fluorescence was detected in the 500–550 and 650–700 nm ranges for BCECF and PI, respectively. *S. aureus* 209P cells were preincubated with acetoxymethyl ester of BCECF (10 μM) in Hank’s balanced salt solution for 30 min at 37 °C, exposed to PI (15 μM) and peptide XIII (25 μM) for 15 min in Mueller–Hinton broth, centrifuged gently in order to concentrate the cells (1600× *g*, 5 min, 20 °C), and examined by CLSM. Control cells were subjected to the same procedures but without addition of the peptide.

## 3. Results

### 3.1. LP Fragment Library Construction, Neural Network Training, and Activity Prediction

**Figure 2:**
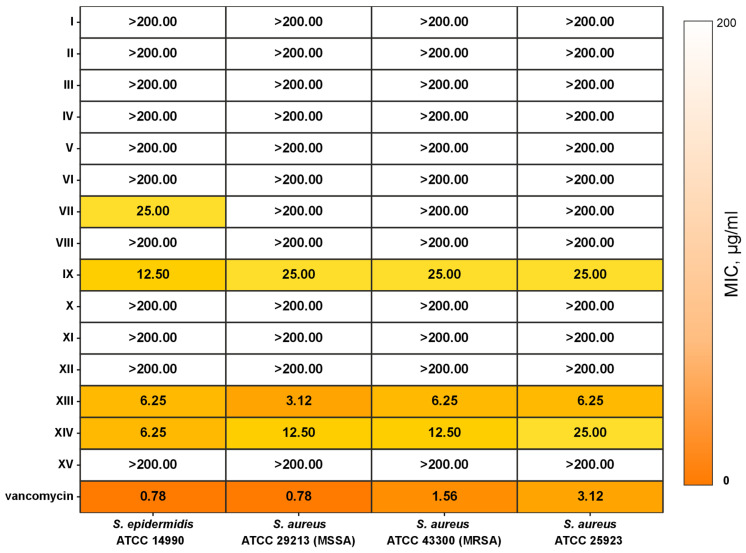
Antibacterial activity of LP fragments against Gram-positive microorganisms.

The workflow of the study comprised the following stages: (1) construction of a spider venom-derived LP fragment library (Figure 1A); (2) assembly of an AMP library for neural network training; (3) application of neural networks to predict LP fragments with potential activity against MRSA (Table S1); (4) validation of the predictions through chemical synthesis and in vitro testing; and (5) investigation of one of the ‘active’ peptides using a combination of high-resolution and wide-line NMR spectroscopy to elucidate the mechanism of activity and selectivity.

The initial step involved compiling a comprehensive dataset of amino acid sequences of LPs derived from spider venom, sourced from existing databases. Over the past decade, the known number of spider venom LPs has increased substantially, from 58 sequences reported in an early review [12] to above 2000 sequences currently available. Rather than analyzing full-length peptides, our focus was on generating peptide fragments of variable lengths (from 9 to 22 amino acids, Figure 1A). The 75,235 LP fragments were generated for the evaluation of antibacterial activity using pre-trained neural networks.

Three neural network-based programs employing different architectures were utilized to predict the activity of LP fragments against MRSA: UniDL4BioPep [29], AMP_Species_prediction [30], and AMPlify [31,32]. Importantly, the prediction of not a generalized antimicrobial activity but an activity against a specific microorganism has been employed. UniDL4BioPep is distinguished by its computational efficiency during both training and prediction phases. Also, this program can be trained on user data. AMP_Species_prediction is capable of predicting activity against multiple microorganisms, including *E. coli* and *S. aureus*. MRSA is not in this list, however if the target of an AMP is the plasma membrane, we suppose that the tested AMP should possess activity against bot *S. aureus* and MRSA. This is because methicillin resistance is not associated with a change in the lipid composition of the plasma membrane but involves other mechanisms, such as alterations in peptidoglycan architecture [33]. AMPlify provides simultaneous predictions of generalized antimicrobial activity and strain-specific activity probabilities based on user-provided peptide datasets. This neural network can be trained using either balanced datasets, containing equal numbers of ‘active’ and ‘inactive’ peptides, or imbalanced datasets, where ‘inactive’ peptides significantly outnumber ‘active’ ones.

Two programs from our set, UniDL4BioPep and AMPlify, allow for custom training on user datasets. The training set must be comprised of AMP composed exclusively of standard amino acids. The ‘active’ peptides were selected according to the following criteria: (1) consisting solely of standard amino acids, containing no disulfide bonds (except for frog skin peptides featuring a single *C*-terminal disulfide bond), and featuring only *C*-terminal amidation as a permissible modification; (2) peptide length does not exceed 50 amino acids; (3) demonstrated activity against at least one MRSA strain with a minimum inhibitory concentration (MIC) below 20 µg/mL; (4) activity determined via standard serial dilution methods. Ultimately, 412 ‘active’ peptides meeting the above criteria were compiled.

During neural network training, ‘inactive’ peptides were also included. For instance, SCMRSA tool used peptides lacking any antimicrobial activity as negative controls [34]. This inactive set was significantly expanded in AMPlify, comprising several thousand sequences [32]. In the later stage, peptides with MIC values exceeding 200 µg/mL against MRSA were selected from the public AMP databases (see above) and incorporated into the pool of ‘inactive’ peptides of the training dataset (in total, 5393 sequences).

After training, we applied the three programs to all compiled LP fragments, providing the corresponding scores for each fragment (see Table S1). This allowed us to select the 15 ‘best’ LP fragments for further study (Table 1).

The following criteria were used: (1) maximum scores generated by the programs for each fragment (Table S1); (2) minimum length; (3) minimum charge; (4) minimum number of leucine residues, which may be a source of an increased hemolytic activity [28]. The programs used to predict peptide activity do not allow to select a variant with *C*-terminal amidation, which, according to the literature data, is important for activity against Gram-positive microorganisms [36]. *C*-terminal amidation increases the overall positive charge of the peptide molecule by +1, which could increase antibacterial activity, but also could lead to unwanted hemolytic activity [37]. Therefore, most of the peptides in our study were amidated. To evaluate the influence of this modification on the antibacterial activity and reduce the overall charge of some highly charged sequences, the *C*-terminus was left free in six peptides (I, II, III, IV, VII, and X). One pair of the peptides (IX and X) were identical, differing only in their *C*-terminal modifications (amidated vs. free *C*-terminus, respectively). Peptides VI–XV represent fragments of the Lycosin family of toxins from the spider *Lycosa hispanica* (Table 1). Among these peptides, VII-XII are fragments of Lycosin 9i of varying lengths (Table 1).

The 15 ‘best’ peptides (Table 1) were synthesized using a solid-phase method. They were purified to homogeneity using HPLC. The accuracy of the synthesis was confirmed by determining their molecular weights using MALDI-TOF/TOF mass spectrometry. The physicochemical characteristics of the obtained peptide samples, their purity, and masses are presented in Table S2. Figure 2 presents the results of the peptides’ antibacterial activity testing. Only three peptides (IX, XIII, and XIV) showed satisfactory anti-MRSA activity. We assume that such a small yield of active peptides is related to the presence of peptides active against various MRSA strains in the training dataset. If we focus on the strains used by us, this would significantly decrease the set of active peptides used for neural network training.

### 3.2. High-Resolution NMR Study of Peptide IX

All ‘active’ peptides IX, XIII, and XIV are homologues, amidated, and represent the fragments of corresponding Lycosins (Table 1, and Figure 1B). Peptide IX is composed of only 13 amino acids, with eight residues identical to the *N*-terminal segments of the LyeTx-I/Ib peptides from the spider *Lycosa erythrognatha* (Figure 1B) [10]. Previous NMR studies have characterized LyeTx-I/Ib within DPC [10] micelles, SDS [38] micelles, and TFE [39]. To compare with the results of previous studies, we investigated peptide IX by NMR spectroscopy in aqueous solution and in micelles formed from the anionic lyso-lipid LPPG.

In aqueous solution, peptide IX seems to be disordered, independent of pH and temperature. First, indication of that was obtained via diffusion measurements. Indeed, the peptide’s translational diffusion coefficient in water amounted to (2.84 ± 0.02) × 10^−10^ m^2^/s (at 30 °C) that corresponds to a hydrodynamic radius R_H_ of 9.8 ± 0.1 Å. This value is close to the expected radius of 10.6 Å for a disordered peptide of similar molecular weight (M = 1542.88 Da, Table S2) calculated using empiric dependence R_H_ = 0.27 × √M [40] and with radius of 10.4 Å calculated by the HYDRONMR program [41] for the peptide IX in extended conformation. The disorder in the peptide structure also agrees with the observation of the narrow dispersion of ^1^H^N^ signals (<1 ppm) and absence of medium and long-range contacts in NOESY spectra (Figure S2B).

Obtaining the ^13^C-, ^15^N-chemical shift assignments of the peptide at natural abundance (Figure S3) allowed to estimate the order parameters S^2^ for most of the residues based on the random-coil index (RCI). The corresponding values did not exceed 0.5 (Figure S4A) adding an argument in favor of random coil state. The only indication of local peptide ordering comes from the temperature gradients of the amide protons (Figure S4A). The amide proton of Gly10 exhibits a temperature gradient with a small amplitude, likely due to shielding from the solvent by the aromatic ring of Phe8 or its ring-current contribution to the chemical shift. The secondary chemical shift of the amide proton signal due to an adjacent aromatic group is usually downfield [42], and its temperature gradient may even be positive [43]. Investigation of the peptide in the pH range of 3–8 yielded a pKa value of 6.02 ± 0.05 for the His12 side chain (Figure S2B). This value corresponds well with the value of 6.45 observed for a random-coil peptide in water [44].

Next, we elucidated the structure of peptide IX in LPPG micelles. We suggest this membrane-mimetic medium is mimicking the environment of cytoplasmic membrane of *S. aureus*, including MRSA. It is well known that the plasma membrane of these bacteria is composed mainly of phosphatidylglycerol (PG) phospholipids [45].

Titration of the aqueous peptide solution with the detergent caused signal broadening and chemical shift changes (Figure S5A,B), consistent with the formation of a peptide/LPPG complex. The signals in the 1D ^1^H NMR spectra ceased changing at a detergent-to-peptide ratio (D:P) of 35:1, indicating the formation of a stable complex (Figure S5B). However, it is known that an LPPG micelle without incorporated proteins or peptides contains on average 125 detergent molecules [46]. Therefore, to suppress possible unfavorable peptide-peptide interactions, the structure of peptide IX was studied at a final D:P of 140:1. The translational diffusion coefficient for the peptide/LPPG complex was measured to be (0.68 ± 0.02) × 10^−10^ m^2^/s (at 30 °C), slightly lower than for detergent micelles alone (0.74 ± 0.02) × 10^−10^ m^2^/s. Calculated radii of the “empty” and peptide-loaded micelles were 37.6 ± 1.0 Å and 40.8 ± 1.2 Å, respectively. Assuming that the peptide/LPPG complex has a density identical to that of an ‘empty’ LPPG micelle consisting of 125 detergent molecules, we can calculate that the peptide/LPPG complex contains 133 ± 7 detergent molecules. Note, that the real value may be slightly higher, since the diffusion of the peptide/LPPG complex was measured using detergent resonances and probably include some contribution from the diffusion of ‘empty’ micelles. Thus, the binding of peptide IX does not significantly change the size of LPPG micelle.

**Figure 3:**
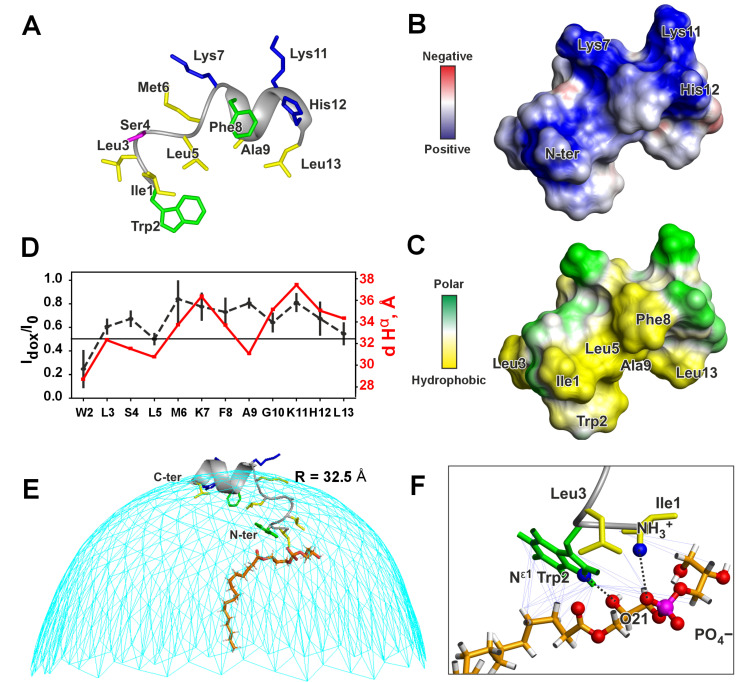
The spatial structure (**A**), molecular surface properties (**B**,**C**), and topology of the peptide/detergent interaction (**D**–**F**) of peptide IX in LPPG micelles. The distributions of electrostatic (**B**) and molecular hydrophobicity [47] (**C**) potentials are shown. (**D**) Signal attenuation in NOESY spectra caused by 16-doxyl stearic acid (black dashed line, left axis) and distances from peptide H^α^ atoms to the center of a spherical micelle (red solid line, right axis). The threshold value of 0.5 distinguishes residues located inside and outside the hydrophobic micelle interior. (**E**) Positioning of peptide IX on the micelle surface (radius 32.5 Å), calculated using attenuation data from panel (**D**). The LPPG molecule is drawn according to the peptide/LPPG complex structure. The terminal methyl group of the fatty acyl chain is assumed to be located in the micelle center. (**F**) NOE-based distance restraints defining the peptide/LPPG complex (see Table S4). Thick dashed lines show hydrogen bonds and salt bridges.

The assignment of ^1^H, ^13^C, and ^15^N resonances of peptide IX in complex with LPPG micelle (Figure S6) allowed us to calculate the secondary chemical shifts of backbone nuclei and estimate the RCI-based order parameters S^2^ for the individual residues, which provide the information about secondary structure and dynamics of the peptide, respectively (Figure S4B). Significant changes in the pKa value of His12 sidechain (to 7.86 ± 0.24, Figure S5C) and amide proton temperature gradients compared to aqueous solution (Figure S4) confirmed the presence of specific peptide-detergent interactions. The NOESY spectra analysis (Figures S6A and S7A) revealed proton-proton contacts characteristic of an α-helix within the Lys7–His12 of the peptide. Combining distance restraints from NOE intensities (τ_m_ of 150 ms) with hydrogen bonding restraints from temperature gradients of amide protons and dihedral angle restraints obtained from secondary chemical shifts allowed us to calculate the spatial structure of the peptide (Figure 3A and Figure S7B, and Table S3). The compact structure features a helix-like segment at the *N*-terminus (Ile1–Met6), followed by a short α-helix (Lys7–Leu13) stabilized by three hydrogen bonds. This structure is supported by low-amplitude amide proton temperature gradients of the residues Lys11–Leu13, indicating that the corresponding HN groups act as donors for the hydrogen bonds (Figures S4B and S7A). In contrast, AlphaFold 3 [16] predicts a fully α-helical structure (Figure S7B).

Analysis of the surface properties of the resulting structure revealed the amphipathic nature of peptide IX in a micellar environment (Figure 3B,C). The *C*-terminal α-helix features amphipathicity. The charged sidechains, Lys7, Lys11, and His 12, along with the *C*-terminal NH_2_ group, form a large polar region. Meanwhile, Leu5, Phe8, Ala9, and Leu13 form a hydrophobic region on the peptide’s surface. The *N*-terminal tripeptide fragment Ile1–Trp2–Leu3 significantly expands this hydrophobic side. The charged *N*-terminal NH_3_^+^ group protrudes into this hydrophobic region (Figure 3B,C).

The localization of peptide IX within the LPPG micelle was examined using the paramagnetic probe 16-doxyl stearic acid, which contains an unpaired electron near the terminal methyl group of the fatty acyl chain. When embedded in the micelle, this unpaired electron is likely positioned within the micelle’s hydrophobic core. To quantitatively evaluate the effect of the paramagnetic probe, the intensities of selected intraresidual NOE cross-peaks for the peptide in LPPG micelles were compared before and after the probe was added (Figure S8). This comparison provided attenuation curve that reflect signal weakening caused by paramagnetic relaxation enhancement, proportional to the proton-electron distance (the closer the distance, the weaker the signal, Figure 3D). Using these data, the approximate peptide position on the surface of the spherical micelle was calculated (Figure 3E, see Section 2.3). The micelle radius that best fits the experimental data (32.5 Å) is much smaller than the radius of the peptide/LPPG complex determined by diffusion measurements (40.8 Å). This suggests that the peptide is deeply embedded within the micelle headgroup region beneath the surrounding aqueous shell.

This model was further refined by incorporating data on the contacts between the peptide and detergent molecule(s) observed in the NOESY spectra (Figure S9, Table S4). These intermolecular contacts involved the side chains of residues Ile1, Trp2, and Leu3, as well as three CH_2_ groups of the fatty acid chain and both glycerol moieties of LPPG (Figure S9). This revealed the presence of a detergent molecule(s) which remains bound to the peptide *N*-terminus for more than 100 milliseconds. Structural calculations using 33 NOE-based distance restraints (τ_m_ of 150 ms) between the peptide and one tightly bound detergent molecule yielded a structure of the complex with a low CYANA target function and few violations of the experimental restraints (Figure S7C, Table S3). Thus, the presence of only one tightly bound LPPG molecule closely matches the experimental data. To obtain realistic orientation of the LPPG molecule, in this calculation, we assumed that the terminal methyl group of the fatty acyl chain (C16) is located in the micelle center. We restricted the distances between this C16 methyl group and peptide H^α^ atoms to the corresponding distances observed in the original model (Figure 3D). The resulting complex is shown in Figure 3E,F. The side chains of Ile1, Trp2, and Leu3 form an interface that interacts with the headgroup of the LPPG molecule; and the Trp2 side chain also contacts the beginning of the fatty acid chain. In addition to van-der-Waals and hydrophobic interactions, the complex is stabilized by a salt bridge between the positively charged peptide *N*-terminus and the negatively charged phosphate group of LPPG, as well as by a hydrogen bond between the HN^ε1^ group of the Trp2 side chain and the O_21_ atom attached to the sn-2 carbon of the second glycerol moiety of LPPG.

There are, of course, other detergent molecules surrounding the peptide within the micelle, but they are highly dynamic and do not give rise to NOESY cross-peaks. For example, the large shift in the pKa value of His12 from ~6.0 in water to 7.9 in LPPG micelles suggests an interaction between the positively charged His side chain and the anionic LPPG molecule.

### 3.3. Interaction of Peptide IX with Phospholipid Membranes by Wide-Line ^31^P-NMR

**Figure 4:**
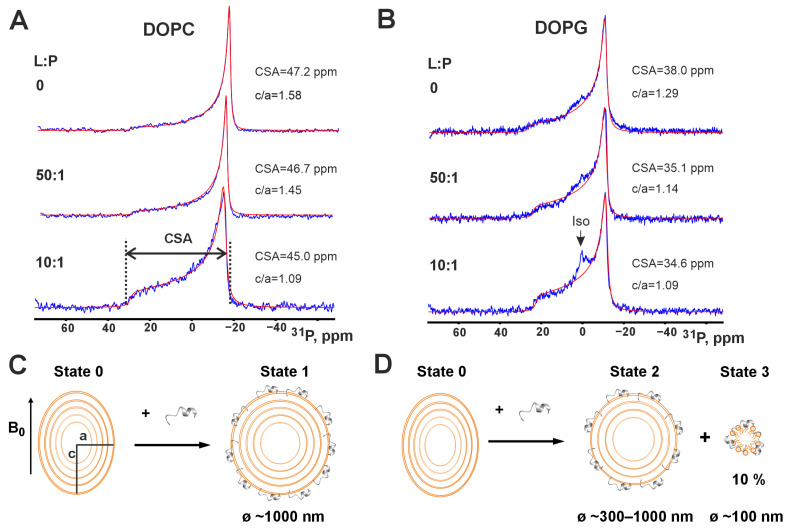
^31^P NMR spectra of MLV DOPC (**A**) and DOPG (**B**) in the presence of peptide IX (25 °C, pH 7). Each panel contain original (blue) and best-fit (red) spectrum. The lipid-to-peptide molar ratios (L:P, indicated on the left) and spectral parameters (anisotropy tensor value on the phosphorus nucleus, CSA, and liposome ellipticity parameter in the magnetic field, c/a) obtained by spectral analysis using the P-Fit [24] program are shown beside the spectra. (**C**,**D**) The schemes illustrate interaction of peptide IX with MLV DOPC (**C**) and DOPG (**D**).

The next step was to investigate the membrane activity of peptide IX by characterizing its interactions with phospholipid liposomes. Multilamellar vesicles (MLVs) composed of either DOPC or DOPG were used as model membranes. DOPC approximates mammalian membranes due to its zwitterionic neutral headgroup, while DOPG, with its negatively charged headgroup, mimics Gram-positive bacterial plasma membranes. Sequential addition of peptide IX to DOPC MLVs induces changes in their ^31^P-NMR spectra (Figure 4A).

The obtained spectra are characterized by an axially symmetric chemical shift anisotropy (CSA) tensor on the phosphorus nucleus. The CSA value for DOPC liposomes in the absence of peptide, approximately 45 ppm, closely matches literature values reported for this temperature (25 °C) [48]. The obtained ^31^P-NMR spectra also allow us to estimate the ellipticity (c/a) of the liposomes under these conditions. The calculated value of approximately 1.6 suggests that the liposomes is likely greater than 1 µm in diameter, considering the dependence of liposome deformability in the spectrometer’s magnetic field on their radius [24]. Increasing the content of peptide IX in the membrane leads to a reduced deformability of the liposomes, as indicated by a decrease in the c/a parameter with decreasing lipid-to-peptide ratio (L:P). For example, c/a decreases from 1.45 at L:P = 50 to 1.09 at L:P = 10 (Figure 4A). This change in liposome deformability likely results from peptide molecules incorporating into the outer monolayer of the liposomes, which increases membrane rigidity and the associated viscoelastic modulus. Nevertheless, even at L:P = 10, the membrane retains its bilayer structure, similar to that observed in the absence of peptide (Figure 4A).

Similar to the DOPC liposomes, the ^31^P-NMR spectra of DOPG liposomes indicate bilayer packing of phospholipid molecules (Figure 4B). The CSA value of 38.0 ppm for liposomes without peptide closely matches the literature value of 37.5 ppm [49]. The size of DOPG liposomes depends on the presence of salt [50]. According to these data, under our conditions without salt, there may be a small fraction of DOPG liposomes with a diameter below of 1 µm [51]. Such liposomes could exhibit averaging of the CSA tensor, which manifests in the spectrum as a broad isotropic signal [52]. This phenomenon appears to be observed under our conditions (Figure 4B, spectra at L:P = 0 and 50).

Unlike zwitterionic membranes made from DOPC, a narrow isotropic signal appeared in the ^31^P-NMR spectrum of anionic DOPG membranes at L:P = 10 (Figure 4B). Decomposing this spectrum into anisotropic and isotropic components, we found that the isotropic signal accounts for approximately 10% of the ^31^P-NMR signal intensity. The induction of the isotropic lipid phase by membrane-active polycationic peptides is typically attributed to the formation of the peptide/lipid aggregates [53,54]. This process occurs in DOPG membranes under the influence of peptide IX. To provide a narrow isotropic ^31^P-NMR spectrum, these peptide/lipid particles should be less than 100 nm in diameter. However, such particles are usually unobservable by high-resolution NMR, indicating that their diameter is larger than 20–30 nm. Thus, they contain large number of the peptide and lipid molecules. Interestingly, 90% of the lipid in the sample remained in bilayer form with reduced ellipticity (c/a ~ 1.09). This suggests that the peptide interacts with the remaining liposomes in the sample, altering the membrane rigidity. Most likely, the peptide disrupts only the outer membrane of anionic MLVs.

Therefore, peptide IX can destabilize anionic phospholipid membranes containing PG, but not neutral membranes composed of PC. This property likely contributes to the peptide’s activity against Gram-positive microorganisms.

### 3.4. Hemolytic Activity of AMP

**Figure 5:**
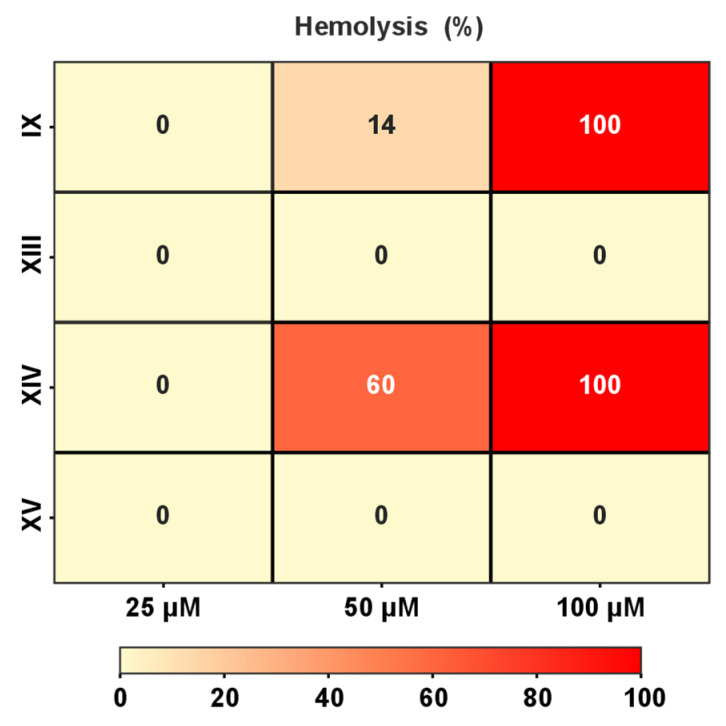
Hemolytic activity of LP fragments against human red blood cells.

In the final stage, we determined the hemolytic activity of the peptides that exhibited antibacterial activity (IX, XIII, and XIV). The ‘inactive’ peptide XV, also *C*-amidated, was used as a negative control. The absence of antibacterial activity in peptide XV (Figure 2) correlates with its lack of hemolytic activity (Figure 5). Contrary to the other two ‘active’ peptides, peptide XIII exhibited minimal hemolytic activity (Figure 5), and, therefore, demonstrated a relatively high therapeutic index, i.e., selectivity toward bacteria. This allows peptide XIII to be used as a lead compound in the development of treatments for infections caused by Gram-positive bacteria, including antibiotic-resistant strains such as methicillin-resistant *S. aureus* (MRSA). It is important to note that peptide XIII is a fragment of the natural toxin Lycosin 6k with only one additional modification: *C*-terminal amidation. This validates the approach proposed in this work.

### 3.5. Mechanism of Antibacterial Action of Peptide XIII

Peptide XIII, which exhibited the highest antibacterial activity (Figure 2) and minimal hemolytic activity (Figure 5), was selected to study the mechanism of its antibacterial action. The mechanism was investigated using the *S. aureus* 209P strain. Before the experiment, the minimum inhibitory concentration (MIC) of the peptide against this strain was measured under conditions identical to those used for testing other peptides (Figure 2), yielding a value of 6.25 μg/mL.

**Figure 6:**
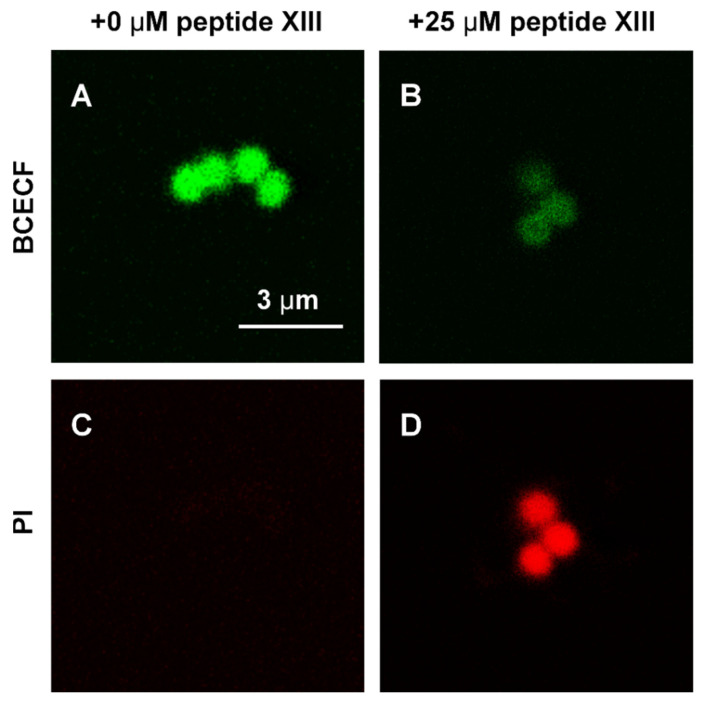
Bactericidal effect of peptide XIII. Confocal images of *S. aureus* bacterial cells stained with BCECF and PI: without peptide addition (**A**,**C**) and treated with peptide XIII at a concentration 25 µM (4-times MIC) for 15 min (**B**,**D**). (**A**,**B**) BCECF fluorescence in the 500–550 nm spectral range. (**C**,**D**) PI fluorescence in the 650–700 nm range. Corresponding images were obtained under analogous conditions and can be compared by signal intensity.

Plasma membrane permeability to fluorescent dyes was assessed by confocal microscopy before and after peptide addition to bacterial cells (Figure 6). The images show that 2′,7′-Bis-(2-carboxyethyl)-5-(and-6)-carboxyfluorescein (BCECF), which is permeable to the plasma membrane and thus making the cells green (Figure 6A) is released from them after 15-min-long exposure of the cells with 25 µM of peptide XIII (Figure 6B). Leakage of the dye results in paler cell color. At the same time propidium iodide (PI) does not penetrate into the living cells making them colorless (Figure 6C). However, after 15-min-long exposure of the cells with 25 µM of peptide XIII PI penetrates into cells making them red (Figure 6D). The entry of PI into cells indicates their death and rupture of the membrane. Meanwhile, the overall form of the cells remains intact. Thus, rapid peptide-induced cell death is caused by permeabilization of the plasma membrane and the formation of pore-like defects. These defects do not affect the overall shape of the cells (Figure 6B,D).

## 4. Discussion

Linear AMP from spider venom with activity against Gram-positive microorganisms are not a novel observation. These include the previously mentioned latarcins [11], lycocitins from the venom of *Lycosa singoriensis* [55], lycosin-II, a peptide isolated from *Lycosa singoriensis* venom [56,57], and peptides LS-AMP-E1, LS-AMP-F1, LS-AMP-G1 from *Lycosa sinensis* venom [58]. The list can be extended by including the antimicrobial peptide GK37, identified through analysis of the venom glands of *Oxyopes forcipiformis* spider [59], as well as peptides PA-Full, PA-Win, and PA-Win2 identified in the transcriptome of the spider *Pardosa astrigera* [30,60]. A new family, HvAMP, consisting of nine homologous members active against various Gram-positive bacteria, was identified from a non-normalized cDNA library of the venom gland of *Heteropoda venatoria* [61].

LyeTx-I, an antimicrobial peptide isolated from the venom of the wolf spider *Lycosa erythrognatha*, was reported in 2010 as a peptide with low hemolytic activity and antibacterial activity against both *E. coli* and *S. aureus* [10]. Several LyeTx-I derivatives were developed, including the shortened 16-residue peptide LyeTx-I mnΔK (Figure 1B). These peptides showed higher activity and an increased therapeutic index (i.e., an improved antibacterial-to-hemolytic activity ratio) than the native peptide [39,62], and exhibited activity against MRSA [63]. LC-AMP-I1, derived from the venom of *Lycosa coelestis*, also demonstrated significant antibacterial effects against MRSA with minimal hemolytic activity [64].

Here, using a novel approach based on analysis of linear fragments of spider peptides with pre-trained neural networks, we identified three peptides with promising activity against MRSA. The structure and membrane activity of one of these molecules, peptide IX, a 13-residue fragment of Lycosin 9i from *Lycosa hispanica* (and *Lycosa erythrognatha*), was investigated by NMR spectroscopy (Figure 3 and Figure 4). The mechanism of action of another molecule, peptide XIII, a 14-residue fragment of Lycosin 6k, was investigated by confocal microscopy. Full-length Lycosins closely resemble LyeTx-I, and peptides IX and XIII resemble its *N*-terminal fragment (Figure 1B).

To establish the structure-activity relationship for antimicrobial peptides (AMPs), information on their three-dimensional structure is essential. For AMPs and membrane-active toxins up to 50–60 amino acids in length, NMR spectroscopy is the most informative structural method. However, the choice of model membrane environment is critical for it. For *S. aureus*, the major plasma membrane lipid is anionic phosphatidylglycerol (PG) [65]. Using lyso-PG lipids, the membrane of these bacteria can be modeled. Although anionic, SDS micelles are not suitable for this.

Peptide IX was investigated in this work using a combination of high-resolution and wide-line NMR methods in aqueous solution, detergent micelles, and phospholipid liposomes. In particular, LPPG micelles were employed to determine the peptide conformation at atomic resolution under conditions suitable for high-quality solution NMR spectra, whereas DOPG vesicles were used to characterize peptide–bilayer interactions in a negatively charged membrane environment that closely mimics bacterial plasma membranes. We found that peptide IX is water-soluble but unstructured in aqueous solution. Structuring of the peptide occurs upon its incorporation into micelles of the anionic detergent LPPG. In this environment, the peptide *C*-terminal region (Lys7–Leu13) adopts an amphipathic α-helical conformation, while the *N*-terminal fragment (Ile1–Met6) forms an irregular hydrophobic helix-like structure. Localization studies using paramagnetic probes and NOE contacts between the peptide and LPPG indicate that the *N*-terminal fragment of the peptide acts as a hydrophobic anchor within the micelle under headgroup region, whereas the stable α-helix is localized not so deeply (Figure 3E). Interestingly, the observation of NOE contacts between the *N*-terminal tripeptide fragment (Ile1–Trp2–Leu3) of peptide IX and the fatty acyl chain and headgroup atoms of LPPG revealed the presence of a specific, long-lived peptide/LPPG complex in the micelle (Figure 3E).

**Figure 7:**
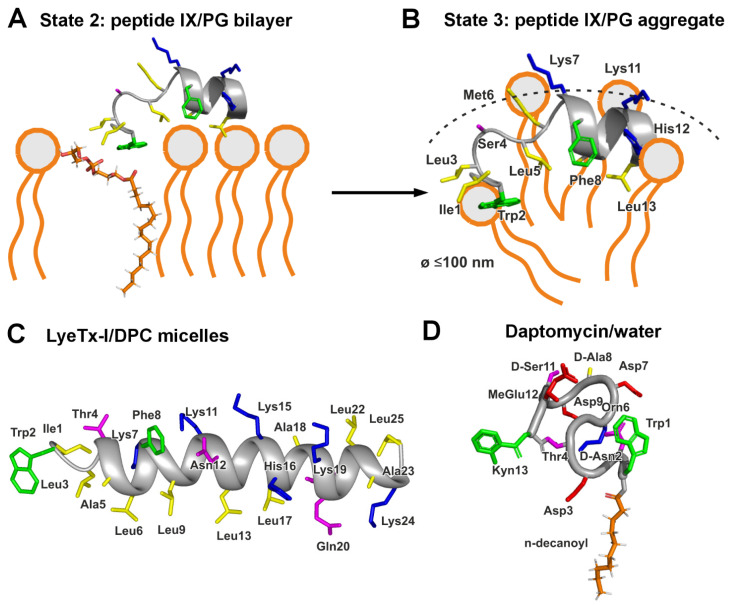
Models of peptide IX interaction with the outer leaflet of anionic PG bilayer (**A**) and peptide IX/PG aggregate (**B**) compared with the structures of LyeTx-I in zwitterionic DPC micelles (PDB ID: 7MMM [10]) (**C**) and cyclic lipoprotein daptomycin in water (PDB ID: 1T5M [67]) (**D**). The complex of peptide IX with one tightly bound lipid molecule (**A**) is represented by the peptide/LPPG complex (Figure 3E,F). Unusual amino acids in daptomycin: ornithine (Orn), (2S,3R)-3-methyl glutamic acid (MeGlu), kynurenine (Kyn), D-amino acids (D-Asp, D-Ala, and D-Ser), and *N*-terminal n-decanoyl.

Intermolecular peptide-lipid NOEs were previously used to map the position of the 19-residue fragment of LL-37 (PepA) within dioctanoyl phosphatidylglycerol (D8PG) micelles [66]. However, the authors used a rather low peptide-to-lipid ratio of 5:1. This state is reminiscent the one formed under conditions of the deterioration of the phospholipid bilayer accompanied with isotropic phase formation (Figure 3B). According to ^31^P-NMR data, peptide IX interacts with membranes composed of DOPC and DOPG, but disrupts only DOPG membranes, inducing an isotropic phase (Figure 4B). Probably the peptide penetrates into the anionic bilayer, forming a complex with DOPG molecules. DOPG and LPPG possess identical head groups. Thus, complex of the peptide IX with DOPG membrane (Figure 4D and Figure 7A, State2) should be similar to the peptide IX/LPPG complex (Figure 3E,F). In this complex, an ionic bond is formed between the peptide’s *N*-terminal NH_3_^+^ group and the lipid’s negatively charged phosphate group. Interestingly, the peptide has two additional NH_3_^+^ groups on the Lys7 and Lys11 side chains, as well as a positively charged His12 side chain (pKa value of ~7.9 in LPPG micelles). Thus, peptide IX has the potential to bind two or three additional anionic lipid molecules. The ability of lipid phosphate groups to form multiple hydrogen and ionic bonds likely determines the tendency to formation of large peptide/lipid aggregates. Thus, as peptide concentration increases on the surface of the anionic membrane, aggregation of the peptide/DOPG complexes occurs, leading to the formation of an isotropic phase observed in the ^31^P-NMR spectra. This phase is interpreted as the peptide/lipid particles with diameters less than 100 nm (Figure 4D and Figure 7B, State3) in which the peptide’s electric charge is neutralized by a stoichiometric number of lipid molecules, resulting in a transformation of their bilayer organization into aggregates with high-curved surfaces (Figure 4D and Figure 7A,B) [23,53].

The same mechanism probably operates on the membranes of Gram-positive bacteria that contain a significant proportion of PG lipids. If the peptide concentration is insufficient for complete micellization of the bilayer, membrane perforation is likely to occur. We hypothesize that the peptide/lipid aggregates spontaneously detach from the bilayer, leaving large pores or defects, which are sufficient to cause cell death while the overall shape of the bacterial cell remains intact. Confocal microscopy illustrates this upon exposure of *S. aureus* 209P cells to peptide XIII (Figure 6).

The effect of peptide IX on DOPC membranes, as measured by ^31^P-NMR, contrasts its effect on DOPG membranes. Changes in the phosphorus CSA tensor and liposome ellipticity value (c/a) upon addition of the peptide (Figure 4A, transition from the upper to the lower spectrum) indicate that the peptide binds to the membrane and increases the viscoelastic modulus of the bilayer, thereby reducing liposome deformation in a magnetic field (a decrease in c/a). However, bilayer disruption and the formation of an isotropic phase do not occur (Figure 4A, lower spectrum and Figure 4C, State1). This likely indicates a less deep penetration of the peptide into the DOPC bilayer compared to DOPG. An indication of this can be found in the structure of LyTx-I, a close homologue of peptide IX (Figure 1B), in zwitterionic DPC micelles [10] (Figure 7C). In case of non-specific peptide/PC interaction this detergent can be a good model. Despite the significant homology between the *N*-terminal region of LyTx-I and peptide IX (8 identical residues out of 13), the 3D structures of the peptides are very different. For peptide IX in anionic LPPG micelles, the α-helix starts at residue Lys7 and the Ile1–Met6 fragment is turned away, allowing the *N*-terminal tripeptide to be immersed in the headgroup region. This conformation is stabilized by the formation of a complex with the LPPG molecule (Figure 7A,B). In contrast, LyTx-I in zwitterionic DPC micelles forms an almost straight helical structure, with an α-helix starting at Thr4 (Figure 7C). This structure is probably formed due to the inability to immerse the positively charged *N*-terminal amino group deep into the zwitterionic micelle. Perhaps a shallower penetration depth of the peptide *N*-terminal fragment into the DPC micelle reflects the inability to form a complex with the zwitterionic detergent.

We hypothesize that the active homologous peptides (IX, XIII, and XIV, Figure 1B) proposed in our study share a similar mechanism of action. The formation of a complex with an anionic lipid appears to be key to the antimicrobial effect of these peptides against Gram-positive pathogens, including MRSA. Forming such a complex serves two purposes: (1) it compensates for the positive charge of the peptide’s *N*-terminus and (2) it makes the *N*-terminus much more hydrophobic. This provides a stronger interaction with the membrane, even for short peptides. Interestingly, both effects can be achieved through *N*-terminal acylation. There are several classes of *N*-acylated peptide antibiotics. The most successful example is daptomycin [67,68] (Figure 7D) and more recent example is gausemycin [69]. These cyclic lipopeptides also demonstrate specific membrane-mediated activity against Gram-positive pathogens and possess a lipid moiety at their *N*-termini crucial for membrane interaction and antimicrobial activity. However, in contrast to linear AMPs which are usually cationic, the molecules of cyclic lipopeptide antibiotics daptomycin and gausemycin are anionic and their activity depends on binding of Ca^2+^ ions [70].

Another example of *N*-acylated AMPs are fungal metabolites—peptaibols and lipo-peptaibols [71]. Similar to the linear spider peptides considered in the present work, peptaibols are also linear and form a helical structure upon contact with the lipid membrane, but unlike spider peptides they are usually uncharged and exhibit broad-spectrum antimicrobial activity and high hemolytic activity with low selectivity. Similar to cyclic lipopeptide antibiotics, peptaibols are formed by non-ribosomal synthesis and contain a high proportion of unusual residues. Interestingly, the acetylation of the *N*-terminus observed in peptaibols also leads to a tilted position of their helices in the lipid membrane or membrane mimetic. This leads to a significant immersion of the *N*-terminus into the hydrophobic region, as observed in the structure of Zervamycin IIB in the DPC micelle [72]. In contrast, the *N*-terminal lipidation observed in lipo-peptaibols confers antimicrobial activity even to very short peptides such as Trichogin GA IV (11 residues) [73]. The structure and topology of the interaction of peptaibols and lipo-peptaibols with lipid membranes has pronounced parallels with the structure and topology of the peptide IX/LPPG complex.

On the other hand, the tight complex of an AMP with an anionic lipid can be considered as a chimeric lipid molecule with a very large polar headgroup represented by the peptide molecule. The overall geometry of the complex promotes the formation of a large positive curvature in the bilayer, effectively leading to membrane micellization. This is in agreement with our ^31^P NMR data and the mechanism proposed above (Figure 4B,D and Figure 7A,B).

Peptides IX, XIII, and XIV demonstrated the highest antimicrobial activity among the 15 peptides tested. All these peptides demonstrate an amidated *C*-terminus, relatively high hydrophobicity (GRAVY > 0.7), and moderate positive charge (+2 ÷ +4) (Table 1). In addition, these peptides are characterized by the presence of the *N*-terminal hydrophobic tripeptides containing aromatic residues (IWL, FWF, and IIW, respectively). The importance of this aromatic tripeptide and possibly other factors is illustrated by the example of peptide V, which also has an amidated *C*-terminus, high hydrophobicity (GRAVY = 1.14), and a positive charge (+3), but lacks the aromatic residues in the *N*-terminal tripeptide ILA and thus remains inactive (Table 1, Figure 2). It is likely that the *N*-terminal aromatic anchor is required for interaction with the PG headgroup and it significantly affects both selectivity and antimicrobial efficacy. Notably, peptide XIII, which exhibits highest antimicrobial activity and lack of hemolysis even at 100 μM, has the highest charge from the selected peptides (+4) and the *N*-terminal tripeptide containing only aromatic residues (FWF). Previously, the important role of the *N*-terminal residues was shown by Feofanov et al. who demonstrated that sequential deletion of the *N*-terminal amino acid residues in the peptide Ltc1-K significantly reduces its toxicity toward eukaryotic cells while simultaneously increasing its antibacterial activity against certain bacterial species [74].

We studied the interaction of peptide XIII with *S. aureus* cells. The phospholipid composition of the plasma membrane of the investigated strain (209P) is identical to that of the MRSA strain. The peptide acts rapidly, significantly reducing the bacterial population within 15 min. The mechanism of action is permeabilization of the plasma membrane. Considering that peptide XIII is homologous to peptide IX, it can be assumed that its structural preferences in different membrane environments are similar to those of peptide IX. Therefore, the mechanism of action of peptide XIII against MRSA is likely based on selective interaction with phosphatidylglycerol (PG) phospholipids in the plasma membrane of these bacteria. It should be noted that peptides IX, XIII, and XIV, active against Gram-positive bacteria, exhibited weaker activity against Gram-negative bacteria (Table S5). Thus, their antibacterial activity spectrum contrasts one of the broad-spectrum AMP, which are generally highly hemolytic (e.g., [54]). Further studies may optimize the activity of peptide XIII and adapt it for in vivo use.

## 5. Conclusions

Using neural networks, we identified several fragments of linear spider venom peptides possessing activity against a panel of *S. aureus* strains, including MRSA. One of the active peptides, IX, was studied by high-resolution NMR spectroscopy in aqueous solution and LPPG micelles. Being unordered in the former milieu, the peptide adopts an ordered structure in the micelle. This structure is mediated by the positioning of the peptide molecule in the micelle. The *N*-terminal part (residues 1–6) penetrates the micelle and positively charged *N*-terminus together with the three *N*-terminal residues (IWL), the lipid interacting peptide motif, form a tight complex with the head group of LPPG molecule. The remaining peptide moiety is exposed to the micelle exterior and is helical. Using wide-line ^31^P-NMR spectroscopy we found that the peptide IX deteriorates MLV DOPG. An isotropic phase is observed likely due to capability of the peptide to form a complex with the phospholipid molecules, similar to one detected in the LPPG micelles. The peptide is unable to deteriorate MLV DOPC, likely due to a shallower penetration in the zwitterionic phospholipid bilayer and inability to from a complex with this lipid. A more active peptide XIII exhibited higher anti-MRSA activity and lower hemolytic activity compared to the peptide IX. We ascribe this to a more favorable complex formation with PG lipids and/or shallower penetration in the zwitterionic membrane. In its turn, this is caused by the presence of solely aromatic residues within the *N*-terminal tripeptide part of the peptide XIII (FWF). We hope that the concept of peptide motifs involved in specific interaction with the bacterial plasma membrane lipids will allow more precisely perform structure-activity investigations in AMP and membrane interacting polypeptides. A choice of NMR as a spectral technique capable to detect lipid/peptide complex formation on the time scale up to the hundred of milliseconds is illustrated here.

## Figures and Tables

**Table 1:** Full-length toxins and their fragments with predicted anti-MRSA activity.

№	Parent LP and Fragment Sequences ^1^	Name	Organism	*C*-Term ^2^	Length	Charge ^3^	Gravy ^4^	Uniprot ID/GenBank
I	EA ***GWMKALKEHVEKLNKTGKLK*** NLKPPETDTCSFAANAYKALATIRETIDTLKNKLC	Pardosin 13f	*Pardosa amentata*	–	20	4	−1.01	–
II	AGLRD ***FMKRLISKGKIGKEKLVAF*** IKRVISRVKSR	Pardosin 11d	*Pardosa palustris*	–	19	5	0.05	A0A8D7ZRV4C/AG6443143.1
III	***AIWSSAMQFFIKHLKK*** ENLKKLG	Alopecosin 6c	*Alopecosa marikovskyi*	–	16	3	0.19	A0A8D7ZRX2/CAG6443179.1
IV	***GIKDYLKKMLLKLK*** EKLKSMTS	Peucetin 6 (Peu 6)	*Peucetia striata*	–	14	3	−0.22	A0A8D7ZRT4/CAG6443209.1
V	M ***ILADLIAKLKVRAA*** KVSG	Trochosin 2l	*Trochosa ruricola*	NH_2_	14	3	1.14	A0A8D8EPX9/CAG6443258.1
VI	IWF ***SLMKFAGKHLAKHQLSKMG***	Lycosin 9l	*Lycosa hispanica*	NH_2_	19	5	−0.33	A0A8D8EPN8/CAG6442973.1
VII	IW ***LSLMKFAGKHLAKHQL*** SKMG	Lycosin 9i	*Lycosa hispanica*	–	16	3	0.04	A0A8D7ZRD0/CAG6442955.1
VIII	IW ***LSLMKFAGKHLAKH*** QLSKMG	Lycosin 9i	*Lycosa hispanica*	NH_2_	14	4	0.03	A0A8D7ZRD0/CAG6442955.1
IX	***IWLSLMKFAGKHL*** AKHQLSKMG	Lycosin 9i	*Lycosa hispanica*	NH_2_	13	3	0.71	A0A8D7ZRD0/CAG6442955.1
X	***IWLSLMKFAGKHL*** AKHQLSKMG	Lycosin 9i	*Lycosa hispanica*	–	13	2	0.71	A0A8D7ZRD0/CAG6442955.1
XI	IWLS ***LMKFAGKHLAKH*** QLSKMG	Lycosin 9i	*Lycosa hispanica*	NH_2_	12	4	−0.22	A0A8D7ZRD0/CAG6442955.1
XII	IWLSLM ***KFAGKHLAKH*** QLSKMG	Lycosin 9i	*Lycosa hispanica*	NH_2_	10	4	−0.83	A0A8D7ZRD0/CAG6442955.1
XIII	K ***FWFKALKSVAKFIA*** KQKLKQHLGSE	Lycosin 6k	*Lycosa hispanica*	NH_2_	14	4	0.92	A0A8D8EQY2/CAG6442961.1
XIV	***IIWLPVLKFLASHV*** AMEQLSKLG	Lycosin 5c	*Lycosa hispanica*, *Alopecosa marikovskyi*	NH_2_	14	2	1.64	A0A8D7ZUM9/CAG6443133.1
XV	***IWWTAVKFFGKQL*** AKHQLSKMG	Lycosin 9o	*Lycosa hispanica*	NH_2_	13	3	0.44	A0A8D8EQW4/CAG6442974.1

^1^ The part of parent sequence that corresponds to the fragment peptide is shown in red. The peptide sequences are taken from [9]. ^2^ *C*-terminal modification. ^3^ Net charge at pH 7.0. ^4^ Hydrophobicity of the peptide fragment on the Kyte-Doolittle hydrophobicity scale [35]. The maximum and minimum values on this scale are +4.5 and −4.5 for poly-Ile and poly-Arg sequences, respectively.

## Data Availability

The data supporting the findings of this study are available in the Supplementary Materials of this article. Experimental restraints and atomic coordinates for peptide IX LPPG micelles have been deposited in the Protein Data Bank (http://wwpdb.org/) under accession code PDB 9WXK. The NMR chemical shifts were deposited in the BMRB database (https://bmrb.io/) under accession codes 26375 and 36788 (peptide IX in water and LPPG micelles, respectively).

## Supplementary Materials

The following supporting information can be downloaded at: https://www.mdpi.com/article/10.3390/pharmaceutics17121526/s1, Table S1: Criteria for the selection of peptides; Table S2: Physicochemical characteristics of LP fragments with predicted anti-MRSA activity; Table S3: Statistics for the best CYANA structures of peptide IX in LPPG; Table S4: Intermolecular NOE contacts between peptide IX and LPPG molecule used in the calculation of the structure of their complex; Table S5: Antibacterial activity of linear peptides against Gram-negative microorganisms. Figure S1: NMR spectra of peptide IX in an aqueous solution; Figure S2: pH titration of peptide IX; Figure S3: ^13^C and ^15^N-NMR spectra of peptide IX; Figure S4: NMR data about the secondary structure and backbone flexibility of peptide IX in an aqueous solution; Figure S5: Titration of peptide IX with LPPG; Figure S6: NMR spectra of peptide IX in LPPG micelles; Figure S7: NMR data defining the secondary structure of peptide IX in LPPG micelles; Figure S8: The fragment of 2D NOESY spectrum of peptide IX in LPPG micelles before and after the addition of the paramagnetic probe; Figure S9: NMR data defines the structure of long-lived peptide IX/LPPG complex; Figure S10. Mass spectra and HPLC chromatograms of peptides IX, XIII, and XIV.

## Abbreviations

16-DSA	16-doxylstearic acid
AMP	Antimicrobial Peptide
BCECF	2′,7′-Bis-(2-carboxyethyl)-5-(and-6)-carboxyfluorescein
CLSM	Confocal Laser Scanning Microscopy
D8PG	Dioctanoyl Phosphatidylglycerol
DOPC	Dioleoyl Phosphatidylcholine
DOPG	Dioleoyl Phosphatidylglycerol
DOPE	Dioleoyl Phosphatidylethanolamine
LP	Linear Peptide
LPPG	1-palmitoyl-2-hydroxy-sn-glycero-3-phospho-(1′-rac-glycerol)
MIC	Minimum Inhibitory Concentration
MHB	Mueller-Hinton Broth
MLV	Multilamellar Vesicle
MRSA	Methicillin Resistant *S. aureus*
PC	Phosphatidyl Choline
PG	Phosphatidyl Glycerol
PI	Propidium Iodide
RCI	Random Coil Index
