# Supplementary material for: NMR Unveils Activity Mechanism of Linear Spider Venom Peptide Fragments Selected by Neural Networks Against Staphylococci Including MRSA
Source: Pharmaceutics. 2025 Nov 27;17(12):1526. doi: 10.3390/pharmaceutics17121526 (PMC12736563; doi:10.3390/pharmaceutics17121526)
Supplement: Supplementary file 1 [file pharmaceutics-17-01526-s001.zip › pharmaceutics-3976687-supplementary.docx]

Article

NMR Unveils Activity Mechanism of Linear Spider Venom Peptide Fragments Selected by Neural Networks Against Staphylococci Including MRSA

Pavel A. Mironov ^1,2^, Anna A. Baranova ^1^, Vera A. Alferova ^1^, Natalya S. Egorova ^1^, Anastasia A. Ignatova ^1^,
Alexey V. Feofanov ^1,2^, Zakhar O. Shenkarev ^1,3^ and Peter V. Dubovskii ^1,^*

^1^ Shemyakin-Ovchinnikov Institute of Bioorganic Chemistry, Russian Academy of Sciences, 16/10 Miklukho-Maklaya str., Moscow, 117997 Russia;

^2^ Bioengineering Department, Faculty of Biology, Lomonosov Moscow State University, 1 Leninsky Gory str, Moscow, 119234 Russia;

^3^ Moscow Center for Advanced Studies, 20 Kulakova str., 123592 Moscow, Russia.

***** Correspondence: [dubovskii@ibch.ru](mailto:dubovskii@ibch.ru).

Supplementary Methods

SM.1. Peptide Synthesis

The peptides were synthesized using standard Fmoc solid-phase peptide synthesis (SPPS) on a Tentagel HL resin modified with a trityl linker (Tentagel-TRT). Coupling reactions were carried out with an 8-fold excess of Fmoc-protected amino acids using HATU as the coupling reagent and DIPEA as the base. Each coupling step was performed for 30 minutes, and the initial C-terminal amino acid was attached for 2 hours. After chain assembly, the peptide-resin was washed, dried, and cleaved with a TFA/DTT/H₂O/TIS mixture (150:4:3:0.5, w/w) for 2 hours. The crude peptides were precipitated with diethyl ether, stored at 4 °C for 8 hours, collected by centrifugation, washed, and vacuum-dried. Purification was performed by preparative reverse-phase HPLC on a YMC Actus Triart C18 (10 µm, 30 × 150 mm) column using a 5–55% acetonitrile gradient. Peptide purity (>95%) was verified by analytical UPLC coupled with an ion trap mass spectrometer (Thermo Finnigan LCQ Deca XP Plus). The yield of peptides based on the initial load on the polymer ranged from 15 to 30%.

SM.2 NMR Diffusion Measurements

The numerical factor 3.6 in formula 2 was obtained by performing a Taylor (Maclaurin) series expansion of the analytical expression for the self-diffusion coefficient around $\varphi_{0}$= 0.5718 in the limit of low volume fraction $\varphi$ [27]:

$D\left( \varphi\right)= \frac{D_{0}(1-\frac{9\varphi}{32})}{1+H\left( \varphi\right)+(\frac{\varphi}{\varphi_{0}})/{(1-\frac{\varphi}{\varphi_{0}})}^{2}}$,

$H\left( \varphi\right)=\frac{2b^{2}}{1-b}- \frac{c}{1-2c}- \frac{bc(2+c)}{(1+c)(1-b+c)}$,

$b\left( \varphi\right)={(9\varphi/8)}^{1/2}$,

$$c\left( \varphi\right)=11\varphi/16$$

**Table S1**. Criteria for selection of peptides.

| Peptide number | AMP_Species_Prediction ^1^ | | UniDL4BioPep ^2^ | | AMPlify ^3^ | |
| --- | --- | --- | --- | --- | --- | --- |
|  | *S. epidermidis* | *S. aureus* | *S. aureus* ^4^ | MRSA ^4^ | MRSA ^5^ | AMP-score ^6^ |
| I | 0.28 | 0.74 | 0.97 | 0.75 | 0.99 | 39.637 |
| II | 0.94 | 0.96 | 0.84 | 0.58 | 0.99 | 24.964 |
| III | 0.95 | 0.97 | 0.94 | 0.82 | 0.98 | 20.614 |
| IV | 0.74 | 0.90 | 0.06 | 0.95 | 0.99 | 25.072 |
| V | 0.93 | 0.95 | 0.83 | 0.94 | 0.92 | 17.221 |
| VI | 0.53 | 0.94 | 0.60 | 0.85 | 0.96 | 20.051 |
| VII | 0.89 | 0.97 | 0.59 | 0.71 | 0.85 | 12.809 |
| VIII | 0.60 | 0.96 | 0.08 | 0.67 | 0.99 | 22.108 |
| IX | 0.95 | 0.95 | 0.72 | 0.66 | 0.99 | 27.522 |
| X | 0.95 | 0.95 | 0.72 | 0.66 | 0.99 | 27.522 |
| XI | 0.08 | 0.67 | 0.22 | 0.71 | 0.99 | 23.474 |
| XII | 0.87 | 0.59 | 0.29 | 0.68 | 0.15 | 6.388 |
| XIII | 1.00 | 0.98 | 0.00 | 0.001 | 0.99 | 34.133 |
| XIV | 1.00 | 0.98 | 0.90 | 0.04 | 0.99 | 31.456 |
| XV | 0.99 | 0.94 | 0.82 | 0.07 | 0.96 | 18.128 |

^1^ The program was used in its original form (<https://github.com/bzlee-bio/AMPSpeciesSpecific>). It predicts antimicrobial activity against five bacterial species. The predicted activities against two species are shown. The output values represent the probability of activity; greater values indicate higher predicted activity. The maximum value is 1.0. Note that the program does not support specifying *C*-terminal modifications of peptides.

^2^ The program with a modified activity prediction module was employed. Instead of a binary classifier, it outputs probabilities according to note #1 above.

^3^ The AMPlify program was used in its author-provided form. (<https://github.com/bcgsc/AMPlify>).

^4^ The neural network was trained on datasets consisting of 441 peptides active against *S. aureus* and 412 peptides active against MRSA, with 1147 inactive peptides used as negative controls.

^5^ The probability of activity (minimum among six values) predicted by an imbalanced sub-model of AMPlify trained with a custom dataset of 412 peptides active against MRSA and 5393 inactive peptides.

^6^ Score indicating generalized antibacterial activity; score above 3.01 is considered indicative of activity [31].

**Table S2**. Physicochemical characteristics of the linear peptides obtained by solid-phase synthesis.

| № | Sequence ^1^ | Calculated molecular mass, (Da) | Measured molecular mass, (Da) | Purity, (%) ^2^ |
| --- | --- | --- | --- | --- |
| I | GWMKALKEHVEKLNKTGKLK | 2337.33 | 2338.32 | 95.95 |
| II | FMKRLISKGKIGKEKLVAF | 2192.32 | 2193.33 | 96.57 |
| III | AIWSSAMQFFIKHLKK | 1934.06 | 1935.08 | 97.81 |
| IV | GIKDYLKKMLLKLK | 1819.10 | 1820.11 | 99.16 |
| V | ILADLIAKLKVRAA-NH_2_ | 1492.96 | 1493.93 | 98.96 |
| VI | SLMKFAGKHLAKHQLSKMG-NH_2_ | 2111.15 | 2111.16 | 95.83 |
| VII | LSLMKFAGKHLAKHQL | 1821.04 | 1822.03 | 97.61 |
| VIII | LSLMKFAGKHLAKH-NH_2_ | 1579.90 | 1579.88 | 96.52 |
| IX ^3^ | IWLSLMKFAGKHL-NH_2_ | 1541.87 | 1542.88 | 97.94 |
| X | IWLSLMKFAGKHL | 1542.87 | 1543.87 | 97.31 |
| XI | LMKFAGKHLAKH-NH_2_ | 1379.78 | 1379.79 | 97.95 |
| XII | KFAGKHLAKH-NH_2_ | 1135.66 | 1135.65 | 97.79 |
| XIII ^3^ | FWFKALKSVAKFIA-NH_2_ | 1654.96 | 1654.97 | 98.72 |
| XIV ^3^ | IIWLPVLKFLASHV-NH_2_ | 1634.99 | 1634.98 | 95.02 |
| XV | IWWTAVKFFGKQL-NH_2_ | 1494.80 | 1494.79 | 96.88 |

^1^ *C*-terminal amidation is indicated by the addition of (NH_2_) at the peptide’s *C*-terminus.

^2^ Purity is given as a percentage determined by HPLC analysis.

^3^ Mass-spectra and HPLC chromatograms are given in Figure S10.

**Table S3**. Statistics for the best CYANA structures of peptide IX in LPPG (P:D 1:140).

| Distance and angle restraints | Peptide IX | Complex  peptide IX/LPPG |
| --- | --- | --- |
| **Total NOE contacts** | 109 | 142 |
| Intraresidual | 34 | 34 |
| Sequential (\|i – j\| = 1) | 45 | 45 |
| Medium range (1 < \|i – j\| < 5) | 30 | 30 |
| Long range (\|i – j\| > 5) | 0 | 0 |
| Intermolecular (peptide – LPPG) | 0 | 33 |
| Lower restrains between H^α^ of peptide and C16 of LPPG | 0 | 12 |
| Hydrogen bond restraints (bonds/upper/lower) | 4/8/8 | 6/12/12 |
| Torsion angle restraints | 22 | 22 |
| Angle φ | 12 | 12 |
| Angle ψ | 10 | 10 |
| Total restraints/per residue | 147/11.3 | 200/15.4 |
| **Statistics for calculated structures** |  |  |
| Structures calculated/selected | 200/20 | 200/20 |
| CYANA target function (Å^2^) | 0.84 ± 0.01 | 2.33 ± 0.08 |
| **Violations of restraints** |  |  |
| Distance (> 0.6 Å) | 0 | 0 |
| Distance (0.2 < r ≤ 0.6 Å) | 1 | 7 |
| Dihedral angles (> 1°) | 0 | 0 |
| **r.m.s.d. (Å) overall** |  |  |
| Backbone | 0.10 ± 0.02 | 0.30 ± 0.10 |
| Heavy atoms | 0.59 ± 0.11 | 0.92 ± 0.24 |

**Table S4.** Intermolecular NOE contacts between peptide IX and LPPG molecule used in the calculation of the structure of their complex.

| Peptide IX | LPPG ^1^ |
| --- | --- |
| H^δ1^ I1 | C2’ H, C3’ H |
| H^ε1^ W2 | C2 H, C3 H, C4 H, C1’ H, sn-3 H |
| H^η1^ W2 | C4 H |
| H^ζ3^ W2 | C3 H |
| H^δ1^, H^ε3^, H^ζ2^ W2 | C3 H, C4 H |
| H^δ1^, H^δ2^ L3 | sn-3 H |

^1^ LPPG atoms are denoted according to IUPAC, see Figure S9A.

**Table S5.** Antibacterial activity of linear peptides against Gram-negative microorganisms.

| Identity ^1^ | MIC (μg/ml) | | |
| --- | --- | --- | --- |
|  | *E. coli*, ATCC 25922 | *E. coli*, lptD | *E. coli*, ΔtolC |
| VII | >200 | 12.5 | >200 |
| IX | >200 | 50 | 200 |
| XIII | 50 | 100 | 50 |
| XIV | 200 | 100 | >200 |
| Ampicillin ^3^ | 3.125 | -^2^ | - |
| Nalidixic acid ^4^ | - | 1.56 | 1.56 |

^1^ – See Table 1 for the amino acid sequences of the peptides;

^2^ – Not tested;

^3^ – A semi-synthetic broad-spectrum antibiotic;

^4^ – Antibiotic belonging to the quinolone group. It inhibits DNA replication by inhibiting DNA gyrase activity. Effective against Gram-negative microorganisms.


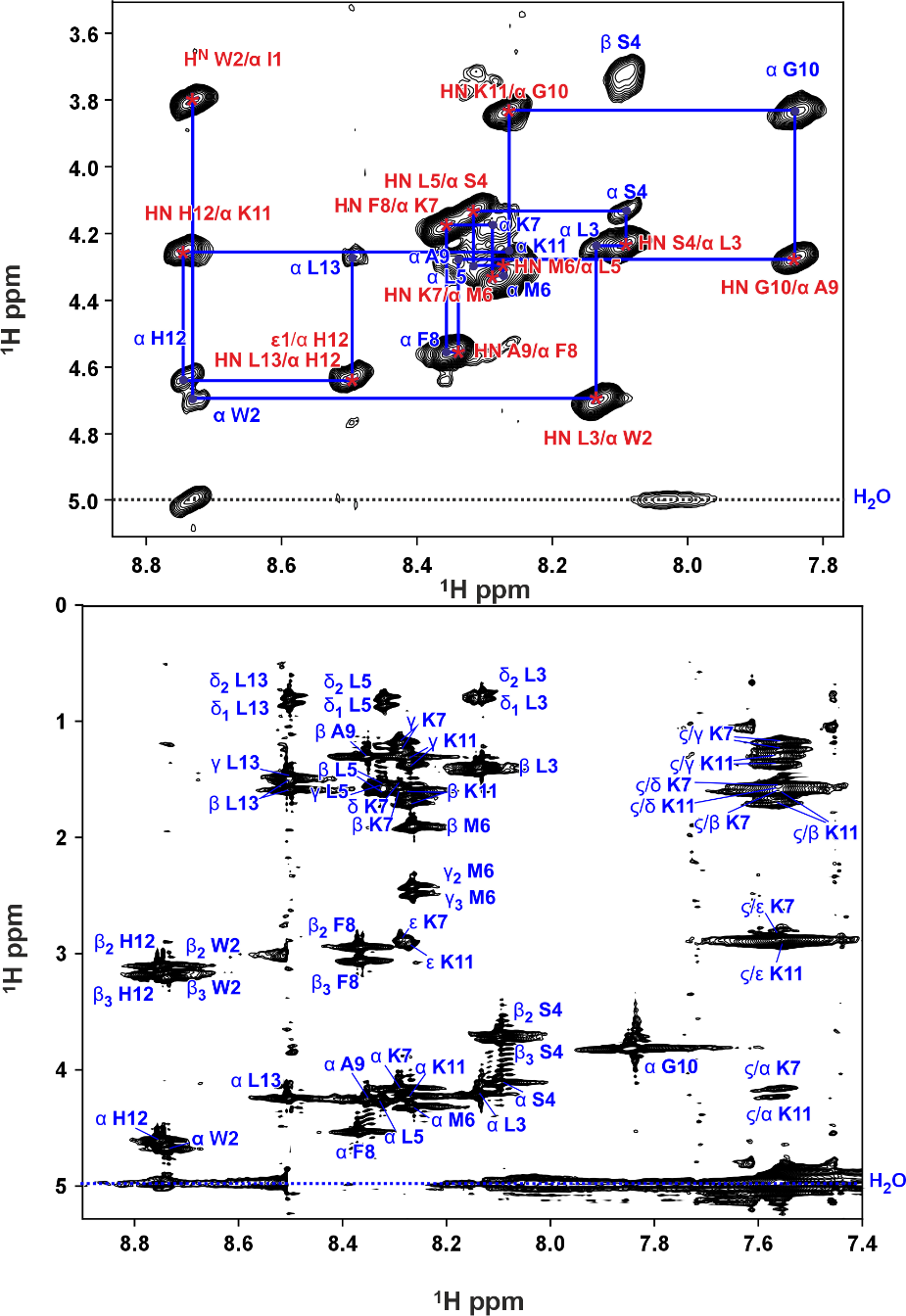


**Figure S1.** NMR spectra of peptide IX in an aqueous solution (1 mM, pH 4.5, 5°C). (**A**) Fragment of NOESY spectrum (τ_m_ = 100 ms). Sequential and intra-residual cross-peaks marked in red and blue, respectively. (**B**) Fragment of TOCSY spectrum (τ_m_ = 80 ms).


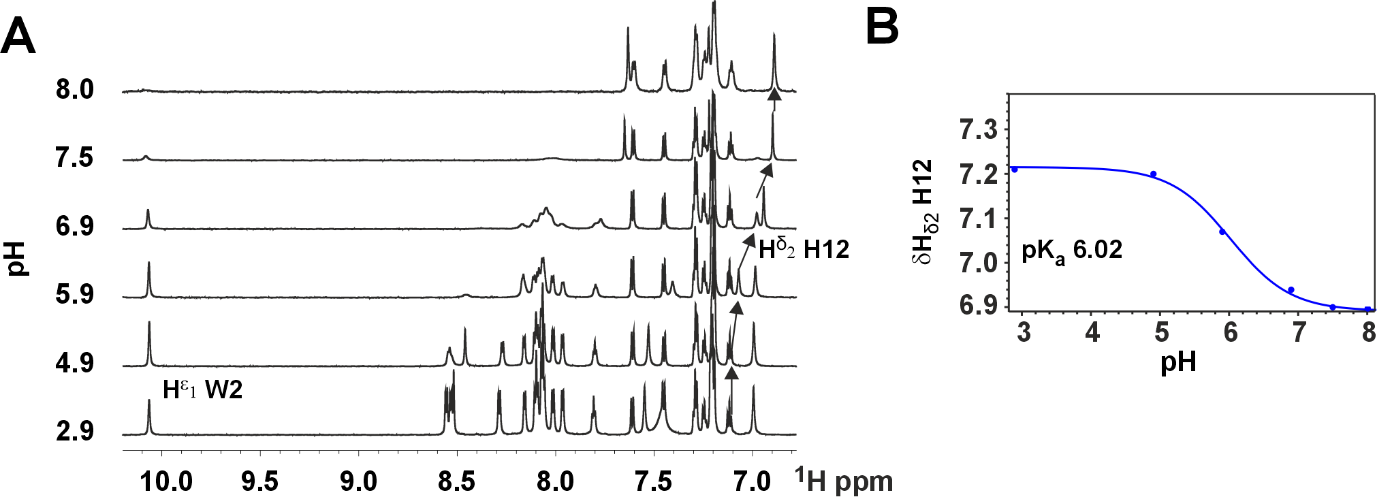


**Figure S2.** (**A**) Amide-aromatic region of the ^1^H 1D NMR spectra of peptide IX in an aqueous solution at different pH (1 mM, 30 °C). (**B**) The dependence of the chemical shift of the H^δ2^ His12 signal on pH is approximated by a standard equation describing the titration of an ionogenic group with pKa = 6.02±0.05.


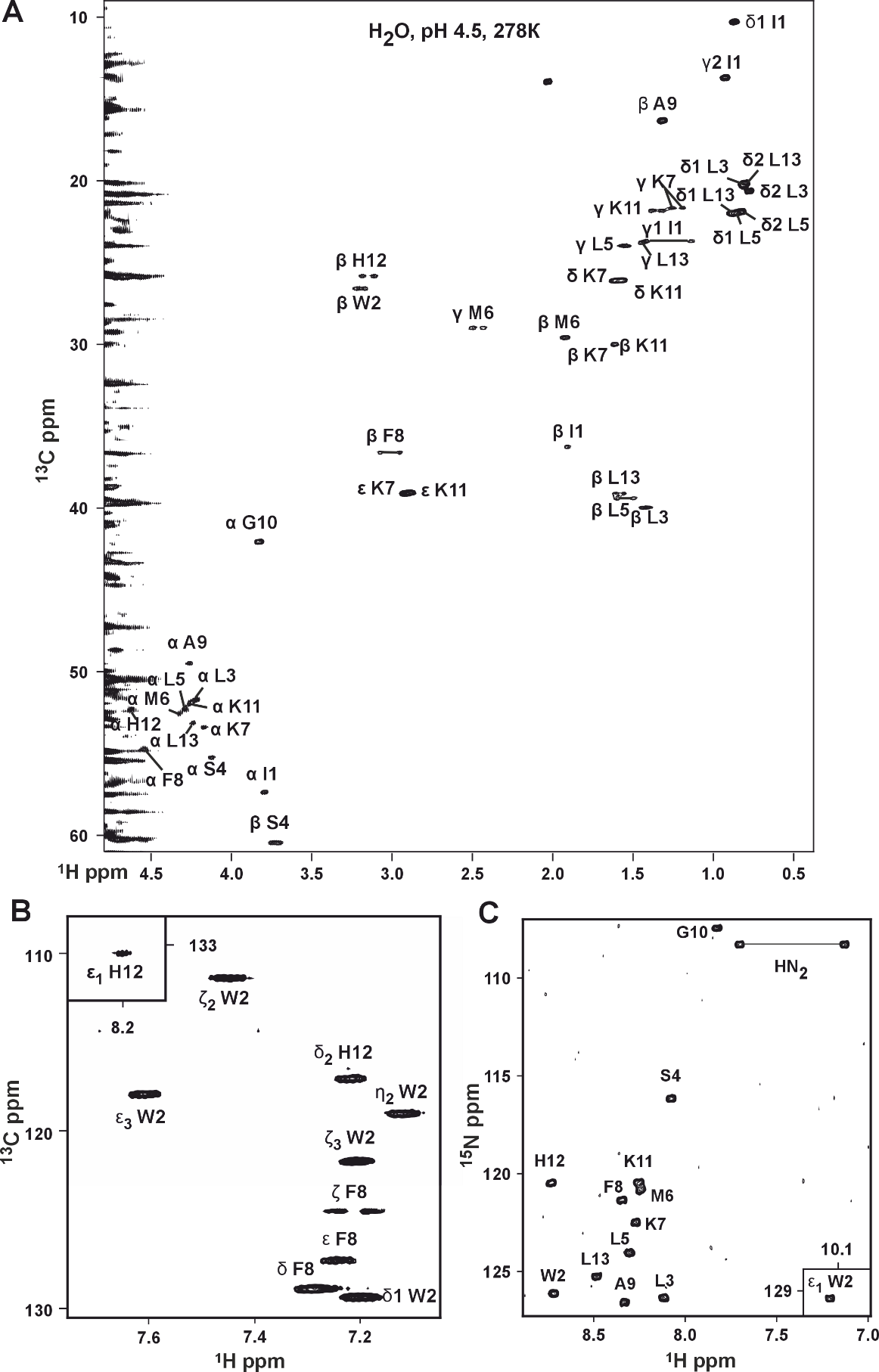


**Figure S3.** ^13^С and ^15^N NMR spectra of peptide IX in an aqueous solution (1 mM, pH 4.5, 5°C). (**A,B**) Aliphatic and aromatic regions of ^13^С-HSQC spectrum. (**C**) ^15^N-HSQC spectrum.


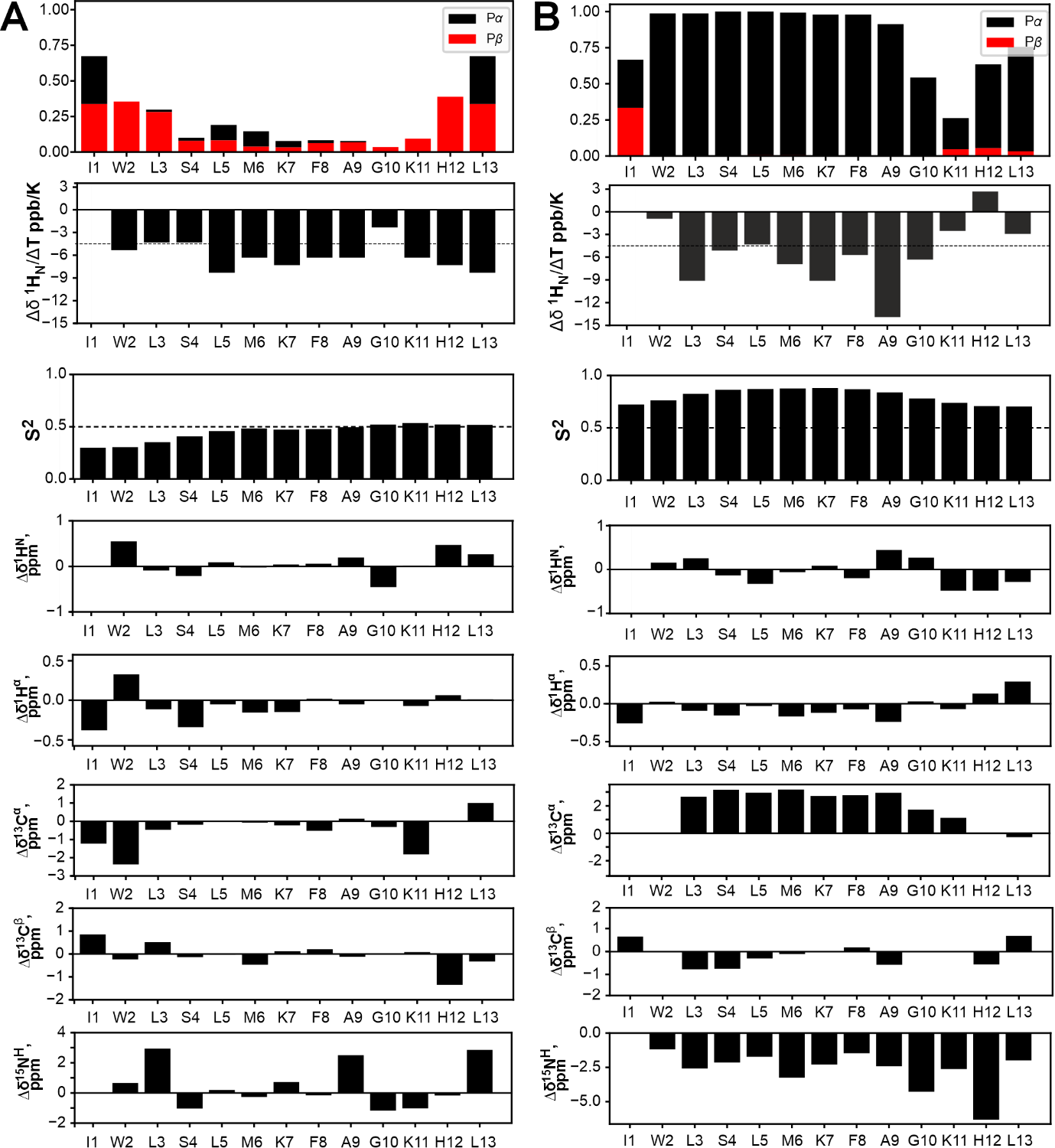


**Figure S4**. NMR data revealing the secondary structure and backbone flexibility of peptide IX in an aqueous solution (**A**) and membrane-mimicking environment of LPPG micelles (**B**). From top to bottom: Probability of α-helical (black) or β-sheet (red) secondary structure for each residue predicted by TALOS-N program. Temperature gradients of amide protons (∆δ^1^H_N_/∆T). The values > −4.5 ppb/K (dashed line) indicate the possible participation of the H^N^ group in hydrogen bond formation. The order parameters S^2^ were calculated using the random coil index (RCI). The secondary chemical shifts of ^1^H^N^, ^1^H^α^, ^13^C^α^, ^13^C^β^, and ^15^N^H^ resonances, that determine the conformation of the peptide backbone.


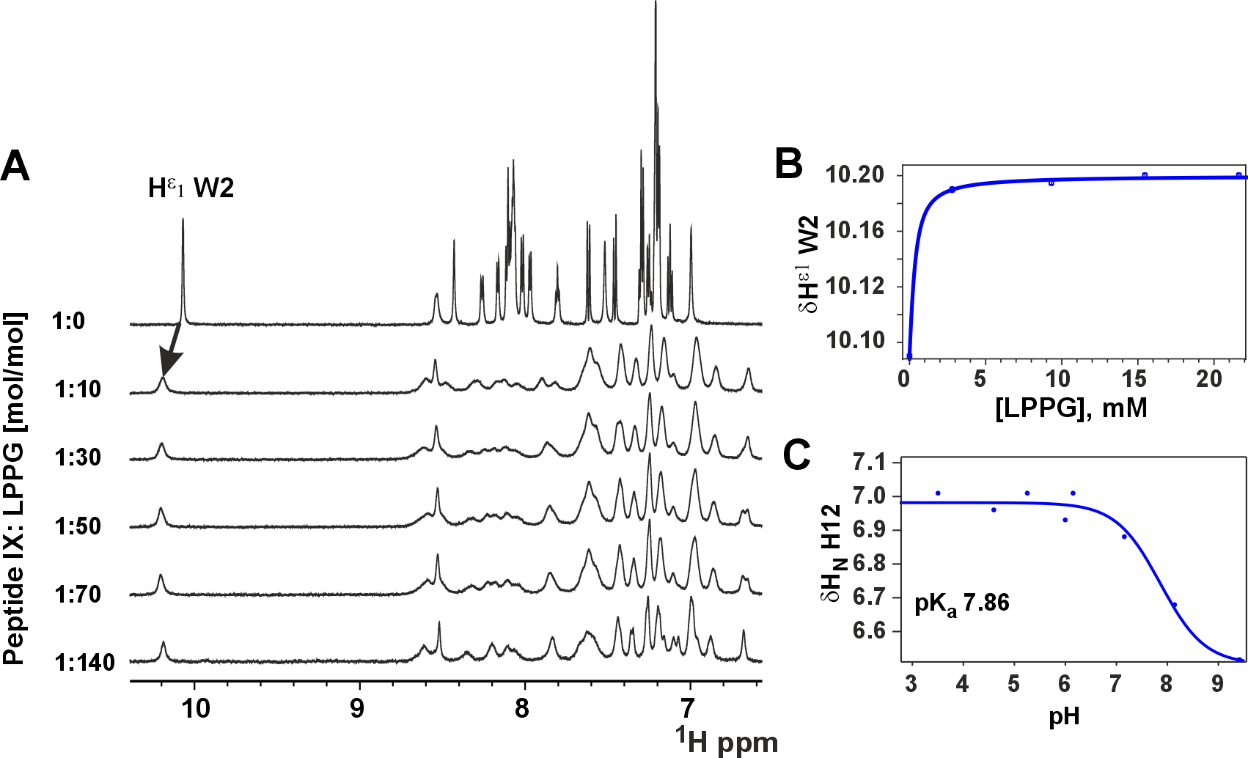


**Figure S5.** (**A**) Titration of 0.12 mM peptide IX with LPPG (pH 4.5, 30°C). Amide-aromatic region of the ^1^H 1D NMR spectra is shown. The indole proton of Trp2 is marked with an arrow. (**B**) The binding curve of peptide IX to LPPG micelles was obtained by modeling how the chemical shift of the Trp indole proton varies with detergent concentration using a partition equilibrium model. (**C**) The dependence of the chemical shift of the H^N^ His12 signal on pH is approximated by a standard equation describing the titration of an ionogenic group with pKa = 7.89 ± 0.24 (P:D 1:140, 45°C).


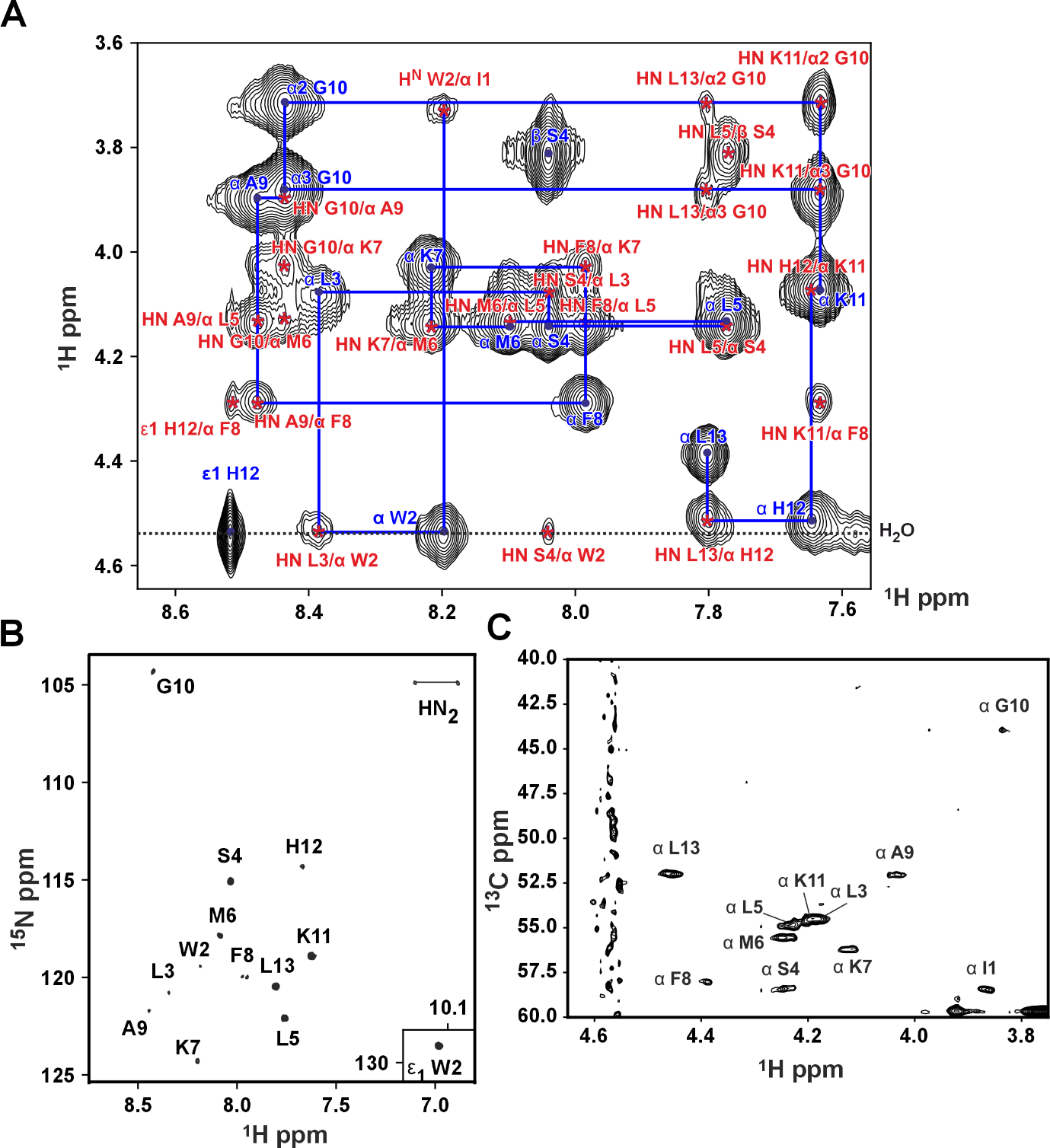


**Figure S6**. NMR spectra of peptide IX in LPPG micelles (1 mM, pH 4.5, 45°C, P:D = 1:140). (**A**) A fragment of the NOESY spectrum (τ_m_ = 150 ms). The sequential and intra-residual cross-peaks are marked in red and blue, respectively. (**B**) ^15^N-HSQC spectrum. (**C**) The C^α^ region of ^13^С-HSQC spectrum.


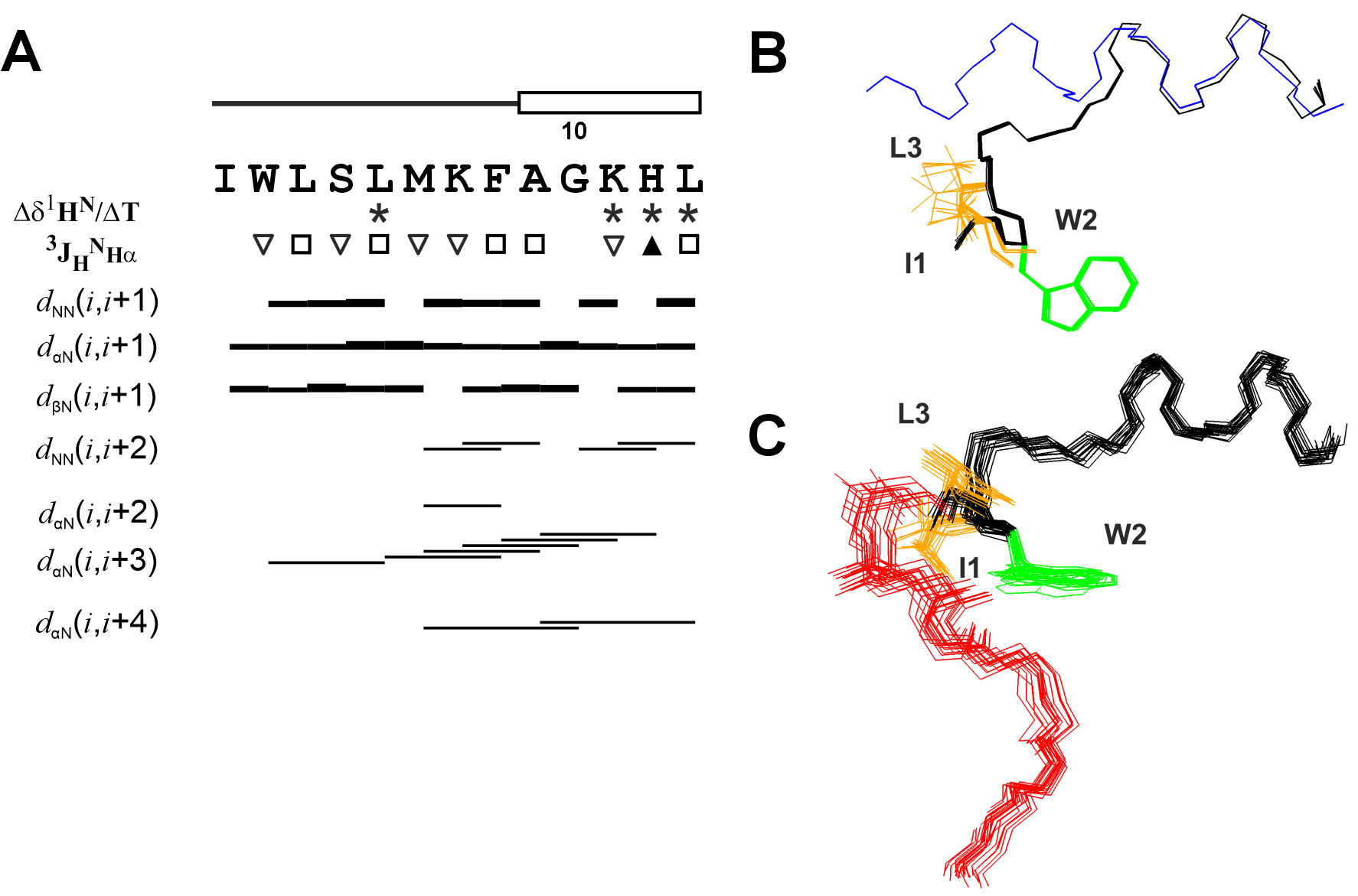


**Figure S7.** (**A**) NMR data defining the secondary structure of peptide IX in LPPG micelles. The residues with the absolute values of the temperature gradients of the amide proton (|Δδ^1^H^N^/ΔT|) lower than 4.5 ppb/K are marked by asterisks. The small (<6 Hz), large (>8 Hz), and medium (others) ^3^JH^N^H^α^ coupling constants are designated by empty triangles, filled triangles, and open squares, respectively. The map of NOE contacts (τ_m_ = 150 ms) is shown, as usual. (**B,C**) Set of the 20 best CYANA structures of peptide IX in LPPG micelles and the peptide IX/LPPG complex, respectively. The peptide backbone is shown in black; AlphaFold model prediction is blue; Ile1, Trp2, and Leu3 sidechains are in orange and green; LPPG molecules is in red.


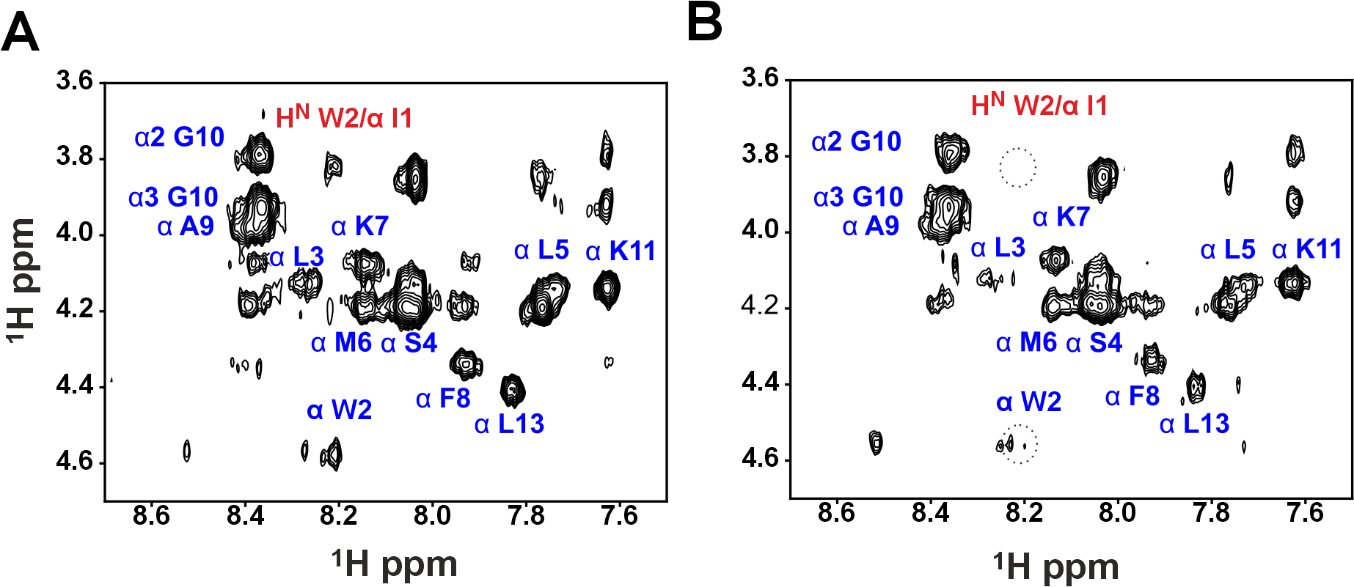


**Figure S8.** The fragments of 2D NOESY spectra (τ_m_ = 100 ms) of peptide IX in LPPG micelles (1 mM, pH 4.5, 45°C, P:D = 1:140) before (**A**) and after (**B**) the addition of the 120 μM paramagnetic probe 16-doxylstearic acid. The signals of residue Trp2 are indicated by the dashed circles, showing a significant decrease in intensity.


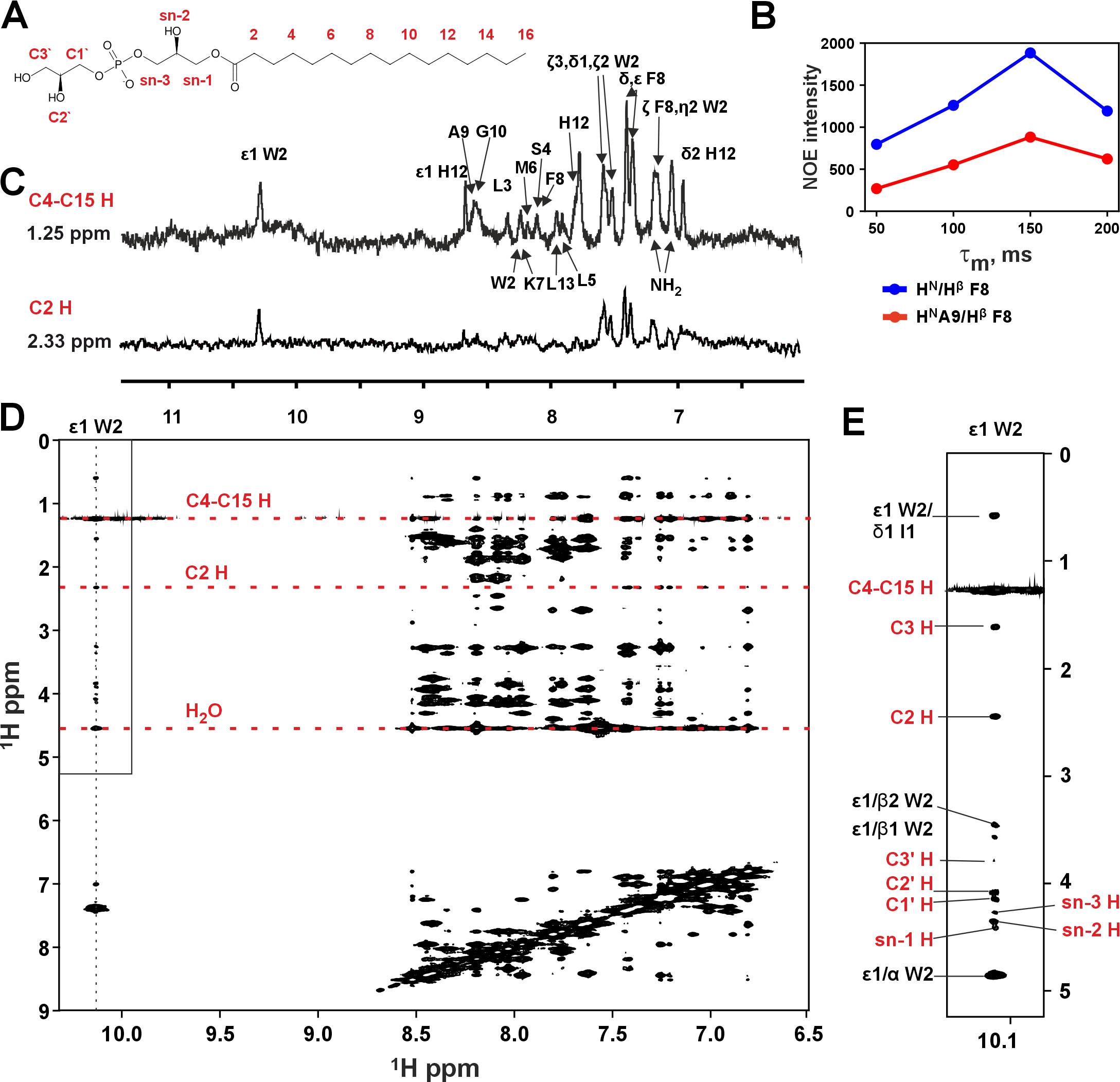


**Figure S9**. NMR data defines the structure of long-lived peptide IX/LPPG complex. (**A**) Chemical structure of LPPG with atom numbering. (**B**) The intensity of NOE signals depends on the mixing time (τ_m_) in the NOESY experiment. (**C, D**) Amide-aromatic region the NOESY spectrum (τ_m_ = 150 ms) of peptide IX in LPPG micelles at 45°C and horizontal slices on the frequency of the acyl chain resonances. Horizontal dashed lines correspond to the chemical shifts of the respective detergent protons and the residual water signal. (**E**) Enlarged region of NOESY spectrum (**D**, boxed) containing cross-peaks to the indole H^Nε1^ proton of Trp2. LPPG protons involved in the intermolecular contacts are marked red.


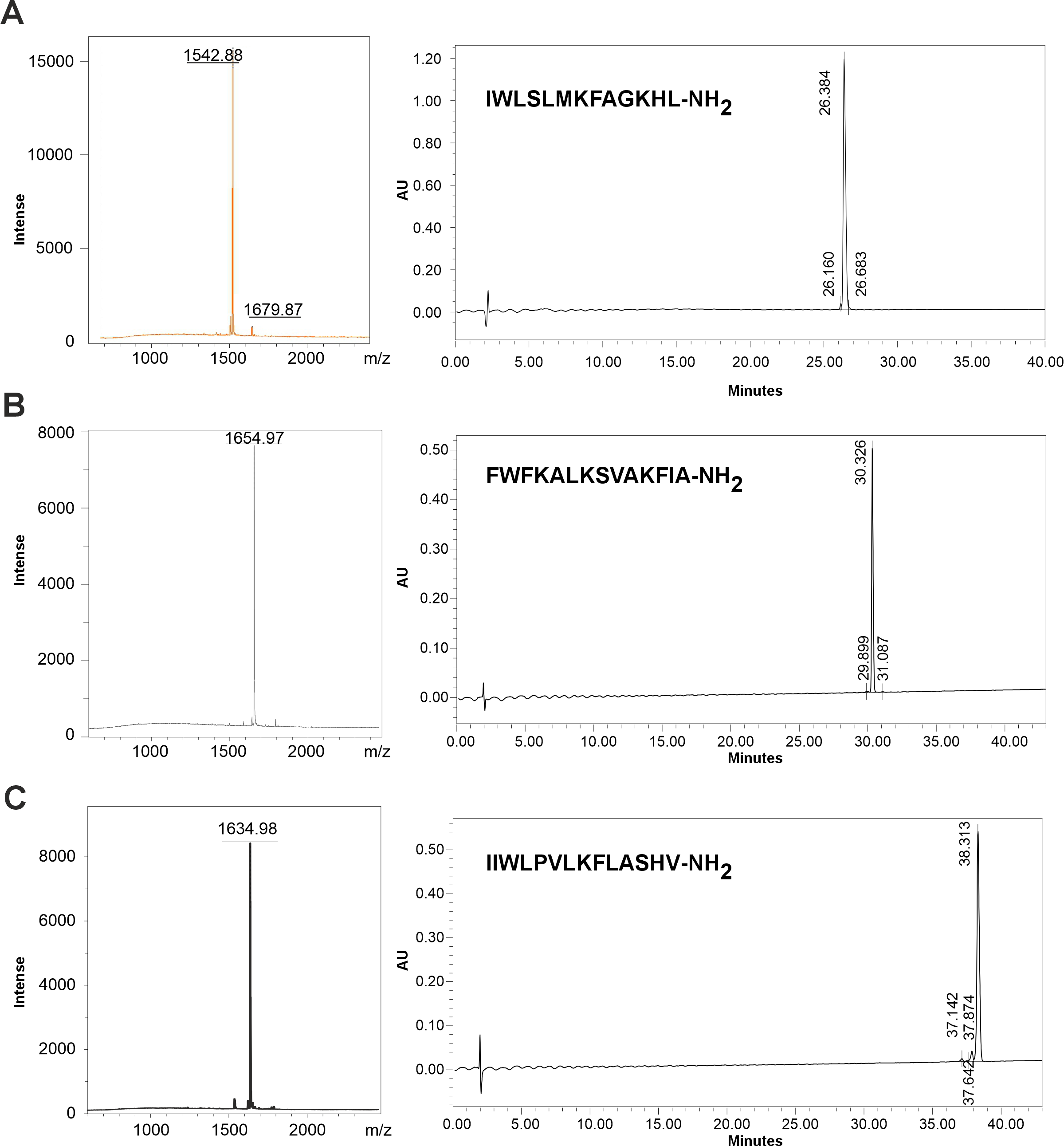


**Figure S10.** Mass spectra and HPLC chromatograms of peptides IX (**A**), XIII (**B**), and XIV (**C**).
